# Antibiotic synergist OM19r reverses aminoglycoside resistance in multidrug-resistant *Escherichia coli*

**DOI:** 10.3389/fmicb.2023.1144946

**Published:** 2023-04-18

**Authors:** Qi Cui, Han-Dong Yu, Qi-Jun Xu, Yue Liu, Yu-Ting Wang, Peng-Hui Li, Ling-Cong Kong, Hai-Peng Zhang, Xiu-Yun Jiang, Anna Maria Giuliodori, Attilio Fabbretti, Cheng-Guang He, Hong-Xia Ma

**Affiliations:** ^1^College of Animal Science and Technology, Jilin Agricultural University, Changchun, China; ^2^Engineering Research Center of the Chinese Ministry of Education for Bioreactor and Pharmaceutical Development, College of Life Sciences, Jilin Agricultural University, Changchun, China; ^3^School of Biosciences and Veterinary Medicine, University of Camerino, Camerino, Italy

**Keywords:** multidrug-resistant bacteria, SbmA, transcription, antimicrobial peptide, PrAMPs

## Abstract

**Introduction:**

The continued emergence and spread of multidrug-resistant (MDR) bacterial pathogens require a new strategy to improve the efficacy of existing antibiotics. Proline-rich antimicrobial peptides (PrAMPs) could also be used as antibacterial synergists due to their unique mechanism of action.

**Methods:**

Utilizing a series of experiments on membrane permeability, *In vitro* protein synthesis, *In vitro* transcription and mRNA translation, to further elucidate the synergistic mechanism of OM19r combined with gentamicin.

**Results:**

A proline-rich antimicrobial peptide OM19r was identified in this study and its efficacy against *Escherichia coli* B2 (*E. coli* B2) was evaluated on multiple aspects. OM19r increased antibacterial activity of gentamicin against multidrug-resistance *E. coli* B2 by 64 folds, when used in combination with aminoglycoside antibiotics. Mechanistically, OM19r induced change of inner membrane permeability and inhibited translational elongation of protein synthesis by entering to *E. coli* B2 via intimal transporter SbmA. OM19r also facilitated the accumulation of intracellular reactive oxygen species (ROS). In animal models, OM19r significantly improved the efficacy of gentamicin against *E. coli* B2.

**Discussion:**

Our study reveals that OM19r combined with GEN had a strong synergistic inhibitory effect against multi-drug resistant *E. coli* B2. OM19r and GEN inhibited translation elongation and initiation, respectively, and ultimately affected the normal protein synthesis of bacteria. These findings provide a potential therapeutic option against multidrug-resistant *E. coli*.

## 1. Introduction

The emergence of multi-drug resistant (MDR) bacterial pathogens is a major threat to global public health (Aslam et al., 2018). At present, due to the extensive use of antibiotics, the family *Enterobacteriaceae* associated with animals have developed multidrug resistance (Van Boeckel et al., 2019). *Escherichia coli* (*E. coli*) is one of the most common pathogens of this family leading to infections in animals and humans with pathogenicity of 10–15% and mortality of 3.8–72%. Usually, aminoglycosides have been used for therapy against *E. coli* infections; however, recent studies have shown that a large number of *E. coli* strains are resistant to aminoglycosides. The resistance rate of aminoglycosides resistant genes and its related phenotypes in *E. coli* from lactating dairy cows have been reported for about 20% in the US (Jeamsripong et al., 2021). Likewise, aminoglycosides resistant bacterial strains of *Enterobacteriaceae* family isolated from different sources in Ireland have also been reported. The bacteria develop different frequencies of aminoglycosides resistance based on their habitat and geographical distribution. The highly exposed areas to antibiotics receives the highest frequency of resistance (Hooban et al., 2022). With the continuous evolution of pathogenic bacteria under the pressure of antimicrobial drugs, the existing antimicrobial drugs are no longer effective in the clinical treatment of bacterial infections; therefore, alternatives to traditional antibiotics are required on priority basis.

Using antibiotic synergists is a promising strategy as it could extend the half-life of existing antibiotics, alleviate the current shortage of effective antibiotics, and reduce the lengthy drug development time and thereby the economic risk. Currently, novel β-lactam antibacterial synergists (Si et al., 2020; Durand-Reville et al., 2021), tetracycline antibacterial synergists (Zheng et al., 2017; Liu Y. et al., 2020), polymyxin antibacterial synergists (Stokes et al., 2017; Song et al., 2021) and macrolides antibacterial synergists (Sturge et al., 2019) have been identified. Furthermore, bee venom enhances the bacteriostatic effect of gentamicin (GEN) and vancomycin (VAN) against methicillin-resistant *Staphylococcus aureus* (MRSA) (Peng et al., 2021). In addition, thiosemicarbazide combined with gentamicin (GEN) prevent Gram-positive bacterial infection by inhibiting DNA gyrase activity by approximately 50% (Wang et al., 2019). However, data on antimicrobial peptides as synergists to restore gentamicin (GEN) sensitivity of Gram-negative bacteria, especially drug-resistant *E. coli*, are limited.

Cationic antimicrobial peptides (AMPs) have also been used as synergists to enhance the antibacterial activity of antibiotics owing to their unique membrane-breaking mechanism (Randhawa et al., 2016; Si et al., 2020; Song et al., 2020; Bai et al., 2021; Zhu et al., 2021). Certain proline-rich antimicrobial peptides (PrAMPs) can directly cross the bacterial cell membrane and exert their antibacterial effects (Roy et al., 2015; Böttger et al., 2016). However, there have been no studies on the use of PrAMPs as adjuvants to improve the antibacterial effect of existing antibiotics. Proline-rich antimicrobial peptide OM19r (VDKPPYLPRPRPIRrPGGr-NH_2_) is a sequence-derived peptide from the existing AMP sequences. The new peptide OM19r of this study was designed by substitution of D-type amino acid at positions 15 and 19 of OM19R (Cui et al., 2021), while OM19R(VDKPPYLPRPRPIRRPGGR-NH_2_) was a hybrid obtained from Oncocin and MDAP-2 fragments (Liu L. et al., 2020). OM19r shown the effective restoration the gentamicin (GEN) sensitivity of multidrug resistant *E. coli*. The effect of OM19r combined with gentamicin (GEN) on the cell membrane and DNA of *E. coli* B2 (mcr-1) was determined *via* a series of fluorescence permeability and DNA binding assays. Meanwhile, an *in vitro* transcription-translation system (TX-TL) was constructed to investigate the effects of OM19r against multidrug resistant *E. coli* B2. Our findings suggest the potential use of OM19r as an aminoglycosides antibacterial synergist to improve the antibacterial activity of aminoglycosides.

## 2. Materials and methods

### 2.1. Bacterial strains and cell culture

Mueller-Hinton (MH) broth and Mueller-Hinton (MH) agar culture medium were purchased from Haibo Biotechnology Co., Ltd. (Qingdao, China) and the antibiotics were purchased from the Institute of China Food and Drug Administration. The bacterial strains were obtained from the Key Laboratory of New Veterinary Drug Research and Development of Jilin Province. The *E. coli* ATCC25922 strain served a quality control. Multidrug resistant *E. coli* B2 (mcr-1) strain was provided by the China Agricultural University while *E. coli* ATCC25922 (ΔSbmA) strain was provided by the Key Laboratory of Animal Production, Product Quality and Security, Ministry of Education. HeLa CellS and mouse macrophage cells (RAW264.7) were cultured in Dulbecco’s Modified Eagle Medium (Gibco) supplemented with 1% (w:v) penicillin–streptomycin and 10% (w:v) heat-inactivated FBS (Sigma-Aldrich) at 37°C in a 5% CO_2_ atmospheric environment.

### 2.2. Experimental animals

BALB/c mice (4 weeks old, weighing 20–22 g) were obtained from the laboratory of Jilin Agricultural University Changchun, China. All the animals were housed in an environment with a temperature of 22 ± 1°C, a relative humidity of 50 ± 1%, and a light/dark cycle of 12/12 h. The laboratory animal usage license (SYXK-2018-0023) was issued by the Laboratory Animal Center of Jilin Agricultural University Changchun, China. All animal studies (including the mice euthanasia procedure) were performed in compliance with the regulations and guidelines of Institutional Animal Care of Jilin Agricultural University Changchun, China.

### 2.3. Antimicrobial activity

The minimum inhibitory concentration (MIC) of antimicrobials was determined by the broth microdilution method (Miao et al., 2020; Zhong et al., 2021). Briefly, the strains to be tested were inoculated into 5 ml of MH broth medium and cultured to logarithmic growth phase (OD_600_ = 0.5), and diluted with fresh MH broth to obtain 1× 10^6^ CFU/ml. The diluted bacterial solution (100 μl) was added to 96-well plates containing different concentrations of antibiotics or different concentrations of antimicrobial peptides. The final concentrations of OM19r strain were 1–128 μg/ml (0.45–57.5 μM). A 100 μl bacterial solution +100 μl MH broth medium served as positive control while 200 μl MH broth medium was kept as negative control. The 96-well plate was incubated at 37°C for 18–20 h, and the MICs were measured at 600 nm. The experiment was conducted in triplicate.

### 2.4. Checkerboard assays

The synergistic antibacterial activity was measured by the checkerboard method (Zhong et al., 2019). Briefly, a 50 μl of diluted OM19r (0–8 μg/ml) and 50 μl of diluted antibiotics (0–512 μg/ml) were put into a 96-well plate, then added a 100 μl of the diluted bacterial solution (1 × 10^6^ CFU/ml) and incubated at 37°C for 16–18 h, and measured the FIC at 600 nm. The experiment was performed in triplicate. The FIC index was calculated as:FIC = MIC (group A combined)/MIC (group A used alone) + MIC (group B combined)/MIC (group B used alone). Where the following were considered as, FIC ≤ 0.5 for synergistic effect, 0.5 < FIC ≤ 1 for additive effect, 1 < FIC ≤ 4 for no effect, and FIC > 4 for antagonistic effect.

### 2.5. Bacterial growth curve and time-kill curve analysis

Growth curves were measured according to the previously described method at OD_600_ nm wavelength at 37°C for 24 h at every 1 h interval (Song et al., 2020). Briefly, different concentrations of OM19r (2 μg/ml), GEN (32 μg/ml), or OM19r combined with GEN (2 + 32 μg/ml) were added to 96-well microplates with an equal amounts of bacterial dilutions (1 × 10^6^ CFU/ml). The time-kill curve was drawn with time as the abscissa and the Log value of the number of colonies as the ordinate (Falciani et al., 2020). Different concentrations of OM19r (8 μg/ml), GEN (128 μg/ml), OM19r combined with GEN (4 + 2 μg/ml) or OM19r combined with GEN (4 + 4 μg/ml) were diluted to contain indicator bacteria (OD = 0.5) in a 1.5 ml centrifuge tube. PBS was kept as a negative control group. After every half an hour, a 100 μl of the diluted bacterial solution was taken out and spread on MH agar medium, and counted after culturing at 37°C for 16–18 h. The procedures were conducted triplicate.

### 2.6. Hemolytic activity

The fresh sheep blood (1–2 ml) was collected at 4°C and centrifuged at 1238 *×* g for 10 min according to a previously described method (Zhong et al., 2020). The collected red blood cells were washed three times with PBS (10 mM, PH = 7.4), and then resuspended in a 9 ml PBS with approximate cell concentration of 10^9^ cells/mL. Erythrocyte suspension (100 μl) was successively added to 96-well plates that were added with antimicrobial peptides (ranging from1 μg/mL to 64 μg/ml) and PBS. After incubation at 37°C for 2 h, the samples were centrifuged at 8800 *×* g for 10 min. The supernatants (180 μl) were transferred to a new 96-well plate, and the absorbance was measured at 570 nm. The absorbance of PBS (100 μl) treated-erythrocyte supernatants (100 μl) served as negative control while the absorbance of 0.1% Triton X-100 (100 μl) treated-erythrocyte supernatants (100 μl) as positive control. Hemolysis was calculated using the following formula:

Hemolysis (%) = [(A _420, peptide_ – A_420, PBS_) / (A_420, 0.2% Triton X-100_ – A_420, PBS_)] * 100.

### 2.7. Cytotoxicity assays

The cytotoxicity of OM19r was determined using 3-(4,5-dimethylthiozol-2-yl)-2,5 diphenyltetrazolium bromide (MTT, United Kingdom) dye reduction test according to a previously described method (Mwangi et al., 2019). Briefly, mouse mononuclear macrophage RAW264.7 and HeLa cells (2.0 × 10^4^ cells/well) were added into 96-well plates at 37°C overnight. Then OM19r was added to the cell cultures to reach a final concentration of 1–64 μg/ml and cultured under a 5% CO_2_ atmospheric environment at 37°C for 24 h. After incubation with MTT (50 ml, 0.5 mg/ml) at 37°C for 2 h, the cell cultures without supernatants were collected by centrifuging at 413 *×* g for 5 min. Subsequently, a 150 μl of dimethyl sulfoxide (DMSO) was added to dissolve the formazan crystals that were created during experiemnt. Absorbance (490 nm) was measured using a microplate reader. Cytotoxicity was calculated using the following formula:

Cytotoxicity (%) = 100 – [(A_570 of peptide treated cells_ / A_570 of control_) * 100].

### 2.8. Membrane permeability assays

The outer membrane changes were measured using 1-N-phenylnaphthylamine (NPN) dye (Ma et al., 2020). NPN is a hydrophobic fluorescent agent normally used for emission of weak fluorescence in aqueous solution but it emits strong fluorescence upon entering to a hydrophobic medium. We added a10 μL of 1 mM NPN solution to each 990 μl of bacterial suspension (1 × 10^6^ CFU/ml) and incubated it at room temperature for 30 min in the dark environment. The groups were as follow: experimental group (Fbos) holding a 100 μl of NPN-containing bacterial suspension +100 μl of antimicrobial peptide mixture, control group (F0) holding a 100 μl of NPN bacterial suspension +100 μl of PBS and control group (F100) holding a 100 μl of NPN-containing bacterial suspension +100 μl of polymyxin B. Fluorescence intensity was detected after every 10 min for 150 min. The mixture was placed in a 96-well dark plate, the excitation wavelength and emission wavelength was adjusted to 350 nm and 420 nm, respectively, and the fluorescence intensity was detected. Each experiment was set up with 3 replicates. Absorption was measured as,

NPN absorption rate = (Fbos-F0)/(F100-F0) × 100%.

The membrane changes were measured using propidium iodide (PI) dye (Mattiuzzo et al., 2010; Mwangi et al., 2019; Ma et al., 2020). Propidium iodide (PI) is a popular red-fluorescent nuclear and chromosome counterstain. Since propidium iodide is not permeant to live cells, it is commonly used to detect dead cells in a population. PI binds to DNA by intercalating between the bases with little or no sequence preference. The bacterial suspension was adjusted to 1 × 10^6^ CFU/ml by the same method mentioned above. First PI dye was added to the bacterial suspension to reach a final concentration of 10 μM. Then PI-containing suspension was mixed at 1:1 with the antimicrobial peptide mixture in a total volume of 200 μl and added to a 96-well dark plate. The excitation wavelength and emission wavelength was set to 535 nm and 615 nm, respectively. Fluorescence intensity was detected after every 10 min for 150 min.

### 2.9. Proton motive force assays

The PMF consists of a pH gradient (ΔpH) and a potential gradient (Δφ), which together constitute an electrochemical gradient (Corbalan et al., 2013). Membrane depolarization was measured using 3,3-dipropylthiodicarbocyanine iodide DiSC3(5) fluorescent dye (Xu et al., 2020). DiSC3(5) was added to the bacterial suspension (1 × 10^6^ CFU/ml) to a final concentration of 0.5 μM, and then the bacterial suspension containing DiSC3(5) was mixed at 1:1 with different concentrations of peptide mixture in a total volume of 200 μl. It was placed on a 96-well light-shielding plate, and the excitation wavelength and emission wavelength was set at 670 nm and 622 nm, respectively. Polymyxin B and PBS served as the positive and negative control, respectively. Membrane depolarization was measured by using BCECF-AM fluorescent dye (Ding et al., 2020). BCECF-AM was added to the bacterial suspension (1 × 10^6^ CFU/ml) to a final concentration of 20 μM, then mixed with the different concentrations of peptide mixture at 1:1 to a total volume of 200 μl, and then added to a 96-well plate protected in dark environment. The excitation wavelength and emission wavelength was set to 522 nm and 488 nm, respectively. The positive control was glucose, and the negative control was PBS, and each group had three replicates.

### 2.10. Efflux pump assays

Fluorescent probe Ethidium bromide (EtBr) was used to detect the effect of drug on non-specific bacterial pumping system and to monitor EtBr efflux from the cell. The bacterial solution was re-suspended in PBS buffer until OD_600_ = 0.5, then a 2 μg/ml EtBr was added, mixed evenly, and incubated in the dark for 30 min. The suspensions containing EtBr in the two experiments were then mixed at 1:1 with antimicrobial peptides to make a total volume of 200 μl. It was placed on a 96-well dark plate, and the excitation wavelength and emission wavelength was set at 600 nm and 530 nm, respectively. The fluorescence intensity was detected after every 10 min for 150 min. The positive control was the efflux pump inhibitor CCCP, and the negative control was PBS, and each group had three replicates (Wang et al., 2015).

### 2.11. Real-time PCR

The qPCR was performed according to a previously described method (Bustin et al., 2009). The bacterial suspension was adjusted to 1 × 10^6^ CFU/ml by the same method as stated. RNA was extracted using RNAiso Plus and the values at 260 nm/280 nm were measured on spectrophotometer. The primers used were SbmA primers (SbmA-F: GAACCTCGAGCTGATCTTCG; SbmA-R: CTGAGCTCCGATTCGAAGG) and internal reference gene primers (rsmC-F: GAAATTCTGGGCGAATACA; rsmC-R: CTTTCACCTCGGAAAAGACG). The reaction system was 10 μl of SYBR Green I Master (2×), 8 μl of ddH_2_O, 1 μl of cDNA upstream and downstream primers, and 0.5 μl of upstream and downstream primers. Reaction program was as follow: pre-denaturation at 95°C for 5 min, denaturation at 95°C for 10 s, annealing at 60°C for 15 s, extension at 72°C for 20 s and a total of 40 cycles. Each experiment was repeated three times. And the fold change of gene expression was calculated by the 2-ΔΔCt method.

### 2.12. Recombinant expression of SbmA

The pET28a- SbmA-6His recombinant expression vector was constructed and transformed to *E. coli* BL21 competent cell for the overexpression SbmA-6His protein. The SbmA protein was purified using Ni-NTA 6FF column. [The process was as follows: Lysed *E. coli* cell with Buffer A (20 mM Tris–HCl pH 7.4, 5% glycerol, 500 mM NaCl. That 6 mM β-Mercaptoethenol, 0.1 mM Benzamidine and 0.1 mM PMSF added immediately before use). Non-affinity protein was washed off by Buffer A added 20 mM imidazole. The target protein was finally eluted by Buffer A added 200 mM imidazole.] The effect of SbmA on the bacteriostatic activity of OM19r against *E. coli* was determined by *in vitro* competitive inhibition assay. The secondary structure of OM19r was constructed by https://zhanggroup.org/I-TASSER/. The structure of SbmA protein was constructed by using the online server Swiss-Model.^1^

### 2.13. Molecular docking

The HDOCK online software was used for molecular docking. The resulting conformations were set to 100 and the scoring function was used to select the conformation with the negative energy. The docking results were visualized by using pymol software.

### 2.14. Confocal laser scanning microscopy

Confocal laser scanning microscopy (CLSM) was performed according to the previously described method (Si et al., 2020). *E. coli* B2 precipitates in logarithmic growth phase (OD_600_ value 0.5) were collected at 2000 × g for 5 min and washed three times with sterile PBS. C-terminal FITC-labeled antimicrobial peptides were added to a final concentration of 0.5 × MIC and1 × MIC, and incubated at 37°C for 30 min. The site of FITC-labeled antimicrobial peptide OM19r entering *E. coli* B2 was observed by confocal laser scanning microscope (FV1000).

### 2.15. Transmission electron microscopy

The intracellular changes in *E. coli* B2 cells induced by OM19r were determined by transmission electron microscope (TEM) according a previously described method (Türkez et al., 2019). The *E. coli* B2 treated with OM19r and Melittin (0.5 × MIC,1 × MIC) were streaked on slides and washed with PBS 3 times, fixed with 2.5% glutaraldehyde for 12 h and 1% osmium for 4 h, then gradient elution was performed with different concentrations (30, 50, 70, 90 and 100%) of acetone, embedded in epoxy resin polymer. Finally, the sections were coated and stained with 2% bisoxyl acetate and lead citrate, and the bacterial samples were observed by using E-1010 TEM (HITACHI JEOL, Tokyo, Japan).

### 2.16. Measurement of total reactive oxygen species

The accumulation of intracellular ROS was measured using DCFH-DA fluorescent dye (Maisuria et al., 2015). DCFH-DA was added to bacterial suspension (1 × 10^6^ CFU/ml) with a final concentration of 10 μM and incubated at 37°C for 30 min. Then centrifuged at 1000 × g for 10 min, resuspended in PBS, and removed the fluorescent probe that did not enter the cells. The bacterial suspension containing DCFH-DA was mixed at 1:1 with the different concentrations of peptide mixture in a total volume of 200 μl and added to a 96-well dark plate. The fluorescence emission was measured by excitation and emission wavelength of 535 nm and of 485 nm, respectively. Hydrogen peroxide and PBS served as positive and negative control, respectively and three replicates were set for each group.

### 2.17. DNA binding assay

The genome of *E. coli* B2 was extracted using genome extraction kit according to manufacturer’s instructions (Shanghai Shangon Biotech Co., LTD., China), and the concentration was adjusted to 100 ng/μL. A 20 μl of peptides and antibiotics with different concentrations were combined with the genome were set in the reaction system. LR_PG_ was used as positive control (Jia et al., 2020). After incubation at 37°C for 1 h, a 2 μl 10 × loading buffer was added and the effect of OM19r on DNA migration was analyzed by 1% agarose electrophoresis.

### 2.18. *In vitro* protein synthesis

*In vitro* protein synthesis was performed according to a previously described method (Garamella et al., 2016). myTXTL® Sigma 70 Master Mix Kit (Daicel Arbor Biosciences, United States) was used to determine the effect of GEN combined with OM19r on protein synthesis *in vitro*. The concentration of OM19r was 0.5 μg/ml and GEN was 0.05, 0.25, 0.5, 1, 2, 5 and 50 μg/ml.

### 2.19. *In vitro* transcription

*In vitro* transcription assays were performed according to a previously described method (Marras et al., 2004). Molecular beacons were purchased from ComateBio, which sequence was: 5′-6-FAM-CCGCGCATCTCGGTTGATTTCTTTTCCTCGGGC GCGG-Dabcyl-3′. The 5′ end was linked to a fluorescent dye (6-FAM), while the 3′–end was coupled to a quencher molecule (Dabcyl). The region which base-pairs with the target rRNA was indicated under the black solid line. Both sides of the end contained repeated GC bases, which were considered the stem of the Molecular beacon, made it quench normally. After mating with the target rRNA sequence, the fluorescence was emitted. OM19r was competitively bound to the target sequence, the molecular beacon configuration did not change and the fluorescence was quenched. The Components of experimental group were as follow: 5 × Buffer (80 μl), NTPs (8 μl), DNA template (4 μl), RNA polymerase (50 μl), RNA inhibitor (1 μl), different concentrations of OM19r (18 μl) and H_2_O replenishment to 200 μl. The Components of negative control group were as follow: 5 × Buffer(80 μl), NTPs (8 μl), RNA inhibitor (1 μl), different concentrations of OM19r (18 μl) and H_2_O and H_2_O replenishment to 200 μl. The Components of positive control group were as follow: 5 × Buffer(80 μl), NTPs (8 μl), 23SrRNA (2 μl), RNA polymerase (50 μl), RNA inhibitor (1 μl), different concentrations of OM19r (18 μl) and H_2_O and H_2_O replenishment to 200 μl. Each group was incubated at 37°C for 20 min. Subsequently, 100% ethanol and 3 M NaAc(pH-5.2) were added, followed by an ice bath for 30 min, and the precipitate was removed by centrifugation at 13523 × g for 10 min at 4°C. After addition of 80% ethanol, the precipitate was removed by centrifugation at 13523 × g for 10 min at 4°C. After vacuum drying for 30 min, a 2 μl molecular beacon was added. Subsequently, a 100 μl of Tris–HCL (pH = 8.8) was added to each well, followed by incubation at 95°C for 2 min and 45°C for 10 min. It was transferred to a 96-well dark plate, and the excitation and emission wavelength was 485 nm and 520 nm, respectively.

### 2.20. mRNA translation

*E. coli* S30 extract translation system(Brandi et al., 2007): The reaction mixture to test standard mRNA translation in bacterial extracts contains, in 30 μl of 10 mM Tris–HCl (pH 7.7): 7 mM Mg acetate, 100 mM NH_4_Cl, 2 mM DTT, 2 mM ATP, 0.4 mM GTP, 10 mM PEP, 0.025 mg/ml PK, 0.12 mM 10-formyl-terahydrofolate, 3 μg/μL tRNA (*Escherichia coli* BL21), an amino acid mixture containing 0.2 mM of all amino acids (with the exception of phenylalanine); 9 μM [^3^H] phenylalanine and 36 μM non-radioactive phenylalanine, an optimized amount of the S30 cell extract (generally 2–6 μl/reaction mixture) and 1–3 μM mRNA (pre-heated 5 min at 65°C). After 30–60 min incubation at 37°C, 20 μl aliquots from each reaction mixture are spotted on 3 mm paper disks which are dropped into 10% ice-cold TCA and processed according to the hot TCA procedure (Mans and Novelli, 1961).

Poly(U) hybrid translation system (Kaminishi et al., 2015): Translation was carried out in Poly(U) hybrid translation system. A 30 μl of buffer containing 3 mM phosphoenolpyruvate (PEP), 0.05 μg/ml pyruvate kinase (PK), 1 mM GTP, 0.15 μg/μL poly(U), 10 μM [^3^H]-Phe-tRNA and 0.2 μM of *Escherichia coli* 30S and 50S were added to the mix after a 5 min incubation at room temperature in the presence of increasing concentrations of OM19r. After 30 min at 37°C the level of poly(U) translation was quantified from the amount of acid-insoluble [^3^H] Phe-tRNA incorporated.

### 2.21. Animal infection model

Mouse peritonitis model: The sample size was selected according to the preliminary infection test (n = 8 per group). Some 6-week-old female mice were intraperitoneally injected with 0.5 ml of *E. coli* B2 (3 × 10^8^ CFU/ml) to establish a mouse peritonitis model. Then after 1 h, different doses of OM19r (8 mg/kg), GEN (8 mg/kg) and OM19r combined with GEN (4 + 2 mg/kg) were injected intraperitoneally. The blank control mice were injected with the same dose of normal saline. Survival of mice treated and colony counts in each organ were recorded over 7 days.

*G. mellonella* infection model: *G. mellonella* larvae were randomly divided into 6 groups (n = 8 per group) and infected with 10 μl of *E. coli* B2 (2 × 10^6^ CFU/ml) in the right hind ventral foot. Then after 1 h, different doses of OM19r (8 mg/kg), GEN (8 mg/kg) and OM19r combined with GEN (4 + 2 mg/kg) were injected intraperitoneally. The blank control mice were injected with the same dose of normal saline. Survival in response to treatment was recorded over 5 days.

### 2.22. Data analysis

All the experimental data were processed by SPSS software using an unpaired two-tailed Student’s *t*-test. Quantitative data were expressed as mean ± standard deviation, *p*-values was considered statistically significant as * *p* < 0.05, ** *p* < 0.01, *** *p* < 0.001, and ns represented non-significant.

## 3. Results

### 3.1. Antimicrobial activity and checkerboard assays

A total of Seven AMPs with good antibacterial activity *E. coli* were synthesized from the existing AMP sequences and evaluated for their efficacy on multiple aspects. OM19r in combination with gentamicin revealed the lowest FIC (FIC = 0.156; Supplementary Table S2) among seven experimental antibacterial peptides. Therefore, a proline-rich antimicrobial peptide OM19r was derived and synthesized (Supplementary Figure S1; Supplementary Table S3). The results of OM19r combined with other antibiotics acting on different sites showed that when combined with gentamicin, the synergistic antibacterial activity was the strongest, and it could restore the sensitivity of multi-drug resistant *E. coli* to gentamicin (Supplementary Table S4). The MIC of OM19r against some Gram-negative bacteria was 1-8 μg/ml (Table 1).

**Table 1:** Minimum inhibitory concentrations (MICs) of OM19r against different bacteria.

Bacterial Species	OM19r (μg/mL)
Gram-negative	
*E. coli* ATCC25922	1
*E. coli* ATCC25922 (△SbmA)	>128
*E. coli* k88	2
*S. enterica* ATCC 14028	2
*S. typhimurium* CMCC50115	2
*S. flexneri* ATCC12022	2
*S. dysenteriae* CMCC51252	2
*K. pneumoniae* CMCC46117	4
Gram-negative (Clinical isolate strains)	
*E. coli* SN5	1
*E. coli* SN3	2
*E. coli* Q12	4
*E. coli* Q14	4
*E. coli* Q45	4
*E. coli* W23	4
*E. coli* QY	4
*E. coli* B2(mcr-1)	4
*S. typhimurium* 1A	4
*A. baumannii* JS1	8
*S. dysenteriae* A3	4
*S. flexneri* RH	8
*K. Pneumoniae* JP20	4
Gram-positive	
*S. aureus* ATCC25923	>512
*S. aureus* (MRSA)	>512
*S. faecalis* ATCC29212	>512
*B. subtilis* ATCC63501	>512

**Figure 1:**
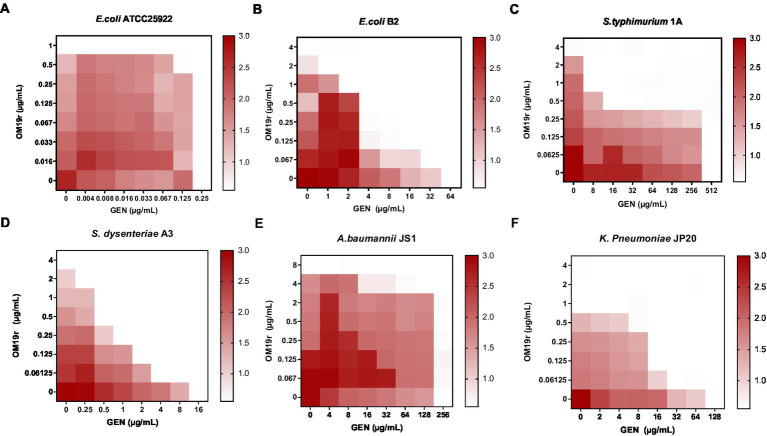
OM19r enhances the antibacterial activity of GEN against some Gram-negative bacteria. **(A–F)** Checkerboard test results of OM19r combined with GEN against *E. coli* ATCC25922, *E. coli* B2, *S. typhimurium* 1A, *A. baumannii* JS1, *S. dysenteriae* A3, and *K. pneumoniae* JP20. The X-axis represents gentamicin 0-MIC concentration, and the Y-axis represents OM19r 0-MIC concentration.

The antibacterial effect of OM19r combined with GEN against drug-resistant bacteria including *E. coli* B2, *S. typhimurium* 1A, *A. baumannii* JS1, *S. dysenteriae* A3, and *K. pneumoniae* JP20 (Supplementary Table S5), was determined using the microbroth dilution method and checkerboard assay. The results showed that OM19r combined with GEN had a synergistic antibacterial effect (FIC ≤ 0.5) against some Gram-negative bacteria (Figures 1A–F). When OM19r was used at a sub-minimum inhibitory concentration (2 μg/ml), the MIC of GEN against resistant *E. coli* B2 decreased from 64 to 1 μg/ml. The FIC of OM19r combined with kanamycin, streptomycin, tobramycin and spectinomycin against drug-resistant *E. coli* B2 was ≤0.5. These results indicate that OM19r combined with aminoglycoside produced synergistic antibacterial effect against drug-resistant *E. coli* B2 (Supplementary Table S6).

### 3.2. Growth curves and time-kill curves of bacteria

**Figure 2:**
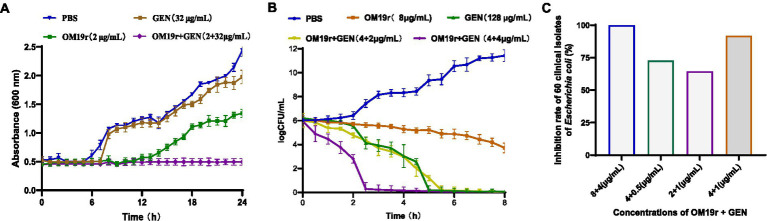
Antibacterial effect of OM19r combined with GEN against *E. coli*. **(A)** 24 h growth curves of OM19r (2 μg/ml) combined with GEN (32 μg/ml) against *E. coli* B2. **(B)** 8 h Time-kill curves of OM19r (8 μg/ml), GEN (128 egg/ml) and OM19r combined with GEN (4 + 2 μg/ml and 4 + 4 μg/ml) against *E. coli* B2. **(C)** Synergistic antibacterial activity of OM19r combined with GEN against 60 clinically isolated *E. coli*. Graphs show means from at least three biological replicates, and error bars indicate standard deviations.

Growth curve analysis showed that OM19r combined with GEN (2 + 32 μg/ml) inhibited the growth of *E. coli* B2, indicating no antagonistic effect between OM19r combined with GEN against *E. coli* B2 (Figure 2A). The time-kill curve of OM19r combined with GEN (4 + 4 μg/ml) showed that *E. coli* B2 could be eliminated within 3 h in a dose dependent manner (Figure 2B). The combined antibacterial activity of OM19r and GEN against 60 other clinical isolates of *E. coli* was also determined. The result showed that OM19r at a low dose (2 μg/ml) combined with GEN (1 μg/ml) inhibited growth of 36 *E. coli* strains (60%) (Figure 2C), while OM19r at a high dose (4 μg/ml) combined with GEN (0.5 μg/ml) inhibited growth of 54 *E. coli* strains (90%) (Figure 2C).

### 3.3. Hemolytic and cytotoxicity assays

**Figure 3:**
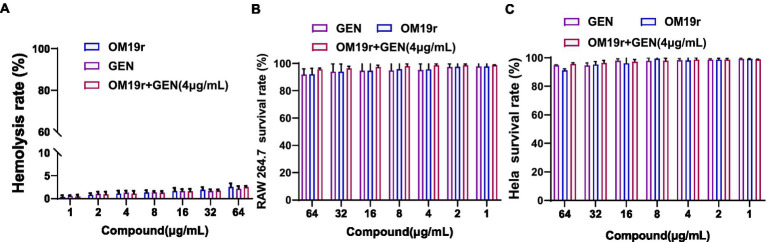
Safety evaluation of OM19r combined with GEN at cell level. **(A)**, Hemolysis of sheep red blood cells treated with OM19r combined with GEN. **(B)** Cytotoxicity of RAW264.7 cells treated with OM19r, GEN and OM19r combined with GEN. **(C)** Cytotoxicity of HeLa cells treated with OM19r, GEN and OM19r combined with GEN. Graphs show mean of three biological replicates, *p*-values were determined using an unpaired two-tailed Student’s t-test.

The hemolysis rate of sheep red blood cells was <5% following treatment with different concentrations of antimicrobial peptide OM19r combined with GEN (Figure 3A). Alternatively, cytotoxicity assays showed that the survival rate of mouse macrophages (RAW264.7) and human cervical cancer cells (HeLa) exceeded 90% after treatment with different concentrations of OM19r combined with GEN (Figures 3B,C).

### 3.4. Membrane permeability

**Figure 4:**
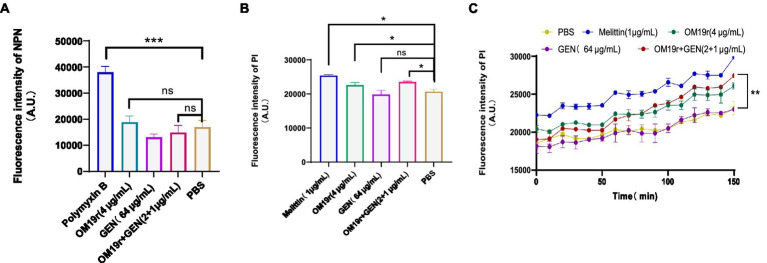
Effect of OM19r combined with GEN on membrane permeability of *E. coli* B2. **(A)** Outer membrane permeability of *E. coli* B2 was measured by NPN probe at 30 min. **(B)**, Inner membrane permeability of *E. coli* B2 was measured by PI probe at 30 min. **(C)** Inner membrane permeability of *E. coli* B2 was measured by PI probe, fluorescence intensity was detected after every 10 min for 150 min. Graphs show mean of three biological replicates, p-values were determined using an unpaired two-tailed Student’s *t*-test.

The results of outer membrane permeability assays showed that OM19r combined with GEN had no effect on the outer membrane permeability of *E. coli* B2 (Figure 4A; Supplementary Figure S2). Meanwhile, inner membrane permeability assays showed that OM19r combined with GEN (2 + 1 μg/ml) increased the inner membrane permeability of *E. coli* B2. There was no significant difference in membrane permeability between OM19r combined with GEN (2 + 1 μg/ml) group and OM19r(4 μg/ml) group (Figures 4B,C). These results indicate that OM19r affected the inner membrane integrity of *E. coli* B2, while GEN had no effect on the cell membrane.

### 3.5. Proton motive force assays

PMF assays results showed no significant differences in △φ and △pH of *E. coli* B2 in OM19r combined with GEN group compared with the negative control group (Supplementary Figures S3A,B).

### 3.6. Efflux pump assays

The fluorescent probe EtBr was used to detect the effects of drugs on the non-specific bacterial pumping system. OM19r combined with GEN did not inhibit the bacterial efflux pump (Supplementary Figures S4A,B).

### 3.7. Real-time PCR and *in vitro* expression of SbmA

**Figure 5:**
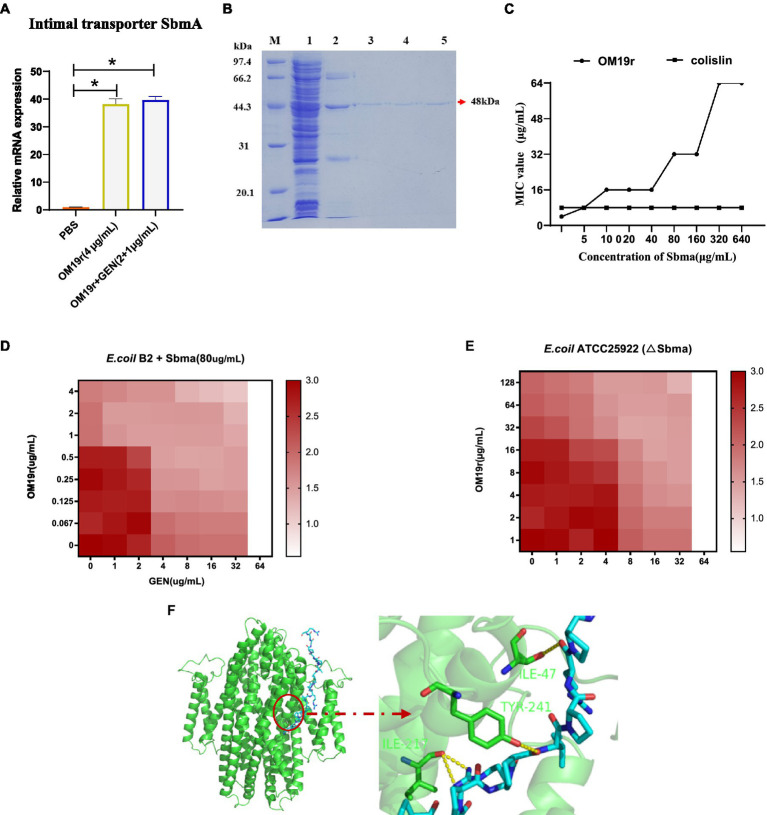
OM19r transported to cytoplasm by the SbmA protein on bacterial inner membrane. **(A)** The mRNA expression of SbmA gene increased after OM19r and OM19r combined with GEN against *E. coli* B2. **(B)** SDS-PAGE results of SbmA protein. M: Premixed Protein Marker(low), 1: flow through 2: 50 mM imidazole wash, 3: 100 mM imidazole wash, 4: 300 mM imidazole first wash, 5: 300 mM imidazole second wash. **(C)** Effect of SbmA on the antibacterial activity of OM19r and colistin against *E. coli* B2 was determined by adding SbmA protein *in vitro*. **(D)** Effect of SbmA on the FIC of OM19r combined with GEN against *E. coli* B2 was determined by adding SbmA protein *in vitro*. **(E)** FIC of OM19r combined with GEN against *E.coli* ATCC25922 (△SbmA) was determined *in vitro*. **(F)** Molecular docking results of both OM19r and SbmA protein binding.

SbmA is a member of the peptide uptake permease family (PUP) and involved in transporting antimicrobial peptides (Armas et al., 2021). The results of qPCR revealed that OM19r combined with GEN upregulated (*p* < 0.05) the mRNA expression level of SbmA in *E. coli* B2 significantly (Figure 5A). But there was no significant difference in SbmA expression between OM19r combined with GEN (2 + 1 μg/ml) group and OM19r(4 μg/ml) group and indicated that SbmA plays a vital role in the entry OM19r to *E. coli* B2. Therefore, SbmA protein (48 kDa) was expressed and purified using *E. coli* expression system, Ni^+^ column and SDS-PAGE (Figure 5B). The effect of SbmA on the antibacterial activity of OM19r was determined *in vitro*. The results showed that the MIC of OM19r against *E. coli* B2 was increased as SbmA protein concentration increased (Figure 5C). In addition, SbmA protein also affected the synergistic antibacterial effect of OM19r combined with GEN against *E. coli* B2 (Figure 5D). The SbmA gene deletion in *E. coli* ATTCC25922 resulted in the loss of the bacteriostatic activity of OM19r(MIC>128 μg/ml) (Figure 5E). Molecular docking showed that OM19r had a good binding affinity with SbmA (binding energy was −262.92 kcal/mol), the binding sites of OM19r and SbmA transporter were ILE-47, ILE-217 and TYR-241 (Figure 5F).

### 3.8. Confocal laser scanning microscopy

**Figure 6:**
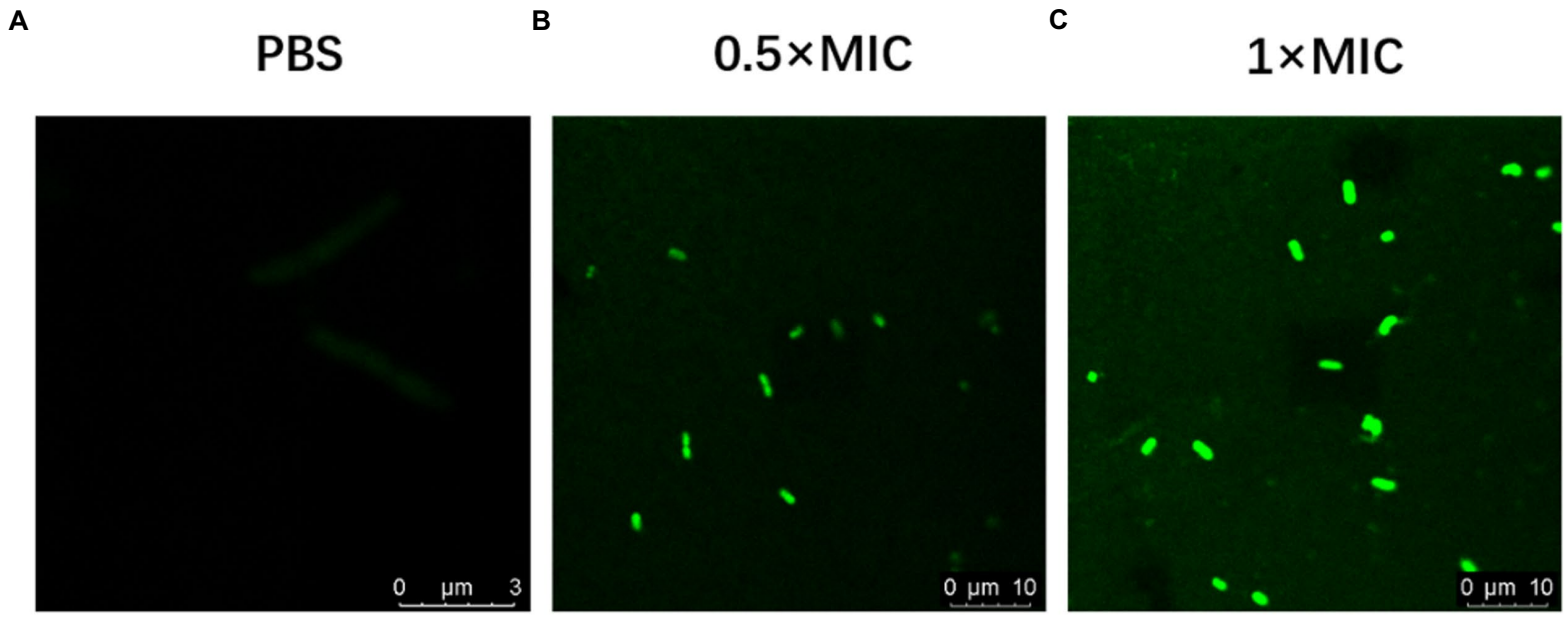
Confocal laser scanning micrograph of *E. coli* B2 treated with OM19r and FITC-OM19r. **(A-C)** Images obtained by fluorescence microscope.

FITC-OM19r in the 0.5 × MIC treatment group displayed a small amount of green fluorescence in the cytoplasm of *E. coli* B2 after 30 min., On the other hand, FITC-OM19r in the 1 × MIC treatment group showed a large amount of green fluorescence in the cytoplasm of *E. coli* B2 (Figures 6A–C). Indicating the binding ability of the antimicrobial peptide OM19r to the cell membrane or accumulation in the cytoplasm.

### 3.9. Transmission electron microscopy

**Figure 7:**
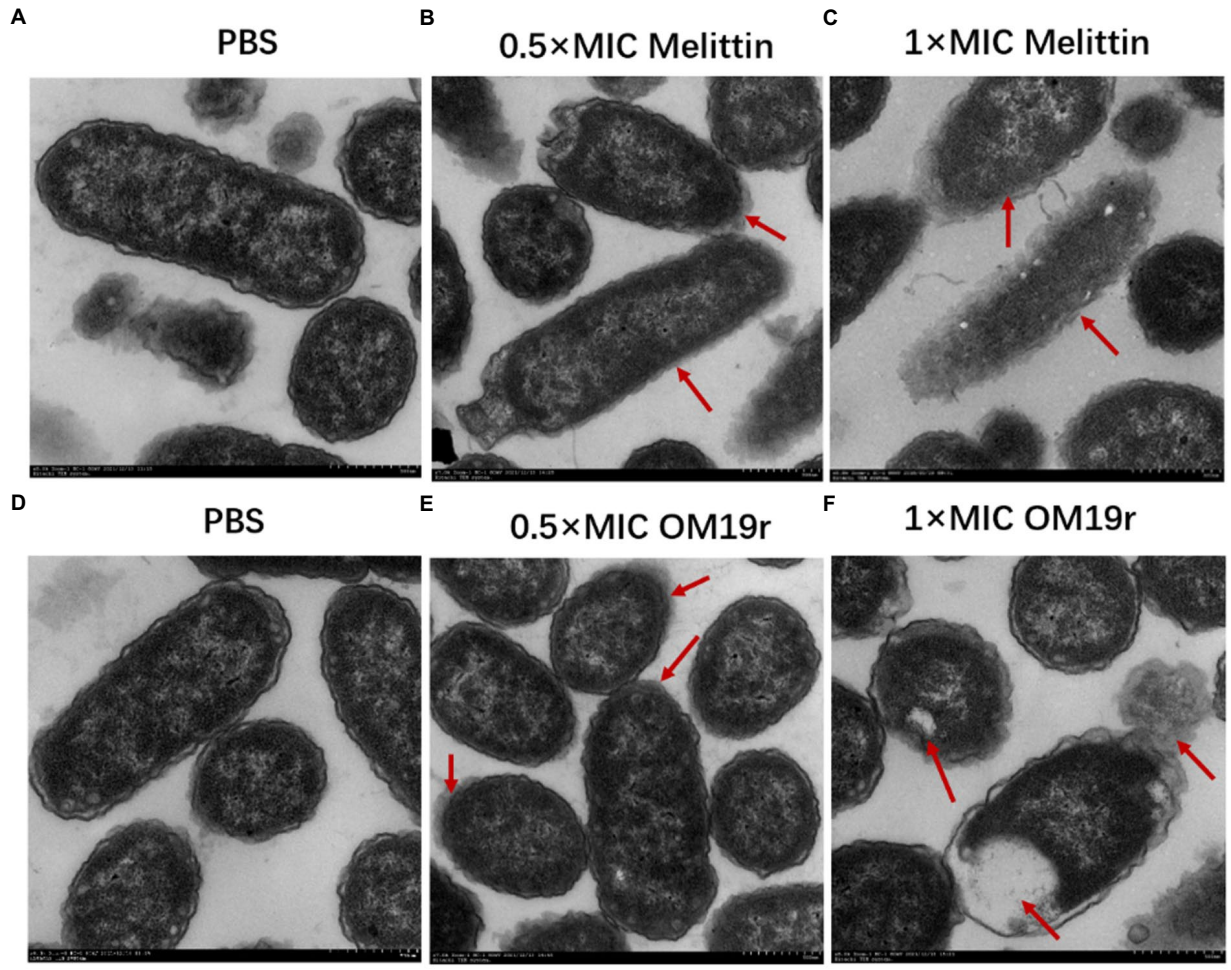
Transmission electron microscopy results of *E. coli* B2 treated with OM19r. (**A)** Transmission electron microscopic images of *E. coli* B2. **(B)** Transmission electron microscopic images of *E coli* B2 treated with Melittin (0.5 × MIC) for 1 h. **(C)** Transmission electron microscopic images of *E coli* B2 treated with Melittin (1 × MIC) for 1 h. **(D)** Transmission electron microscopic images of *E. coli* B2. E，Transmission electron microscopic images of *E coli* B2 treated with OM19r (0.5 × MIC) for 1 h. D, Transmission electron microscopic images of *E coli* B2 treated with OM19r(1 × MIC) for 1 h. Red arrows in B, C, and E points to the cell membrane;The red arrow in F points to intracellular vacuolization.

The TEM results showed that the cell membrane of *E. coli* B2 was smooth, while the cell contents were full prior to OM19r treatment (Figures 7A,D). The membrane of *E. coli* B2 cells treated with Melittin was not clear and the contents were separated (Figures 7B,C). After one hour of OM19r treatment with Melittin at 0.5 × MIC, some contents were leaked and the edge of *E. coli* B2 membrane (Figure 7E). While *E coli* B2 treated for one hour with OM19r at 1 × MIC shown a complete leakage of the contents (Figure 7F). This suggests that OM19r entered bacteria and exerted its antibacterial effect.

### 3.10. Measurement of total reactive oxygen species

**Figure 8:**
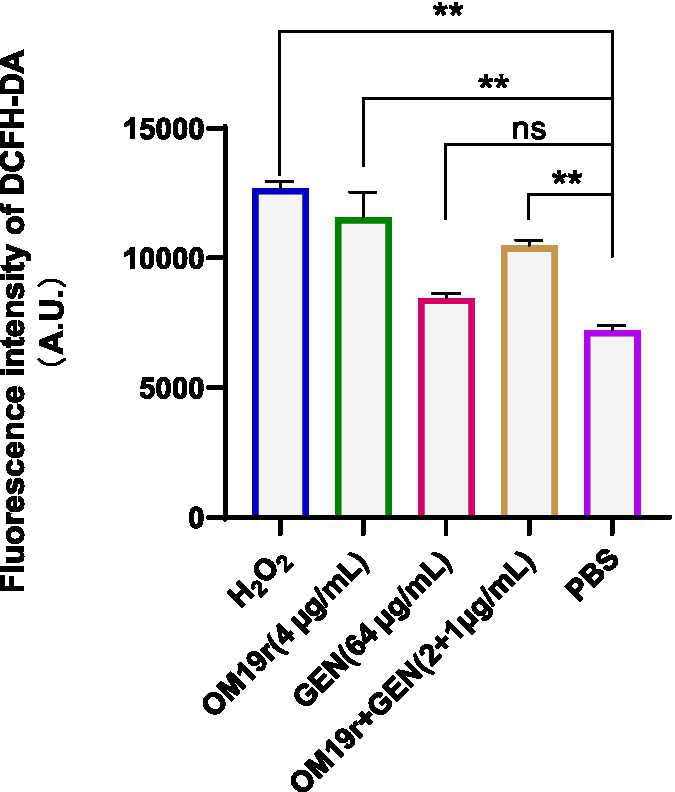
ROS accumulation in *E. coli* B2 cells determined by DCFH-DA fluorescent probe. Graphs show mean of three biological replicates, p-values were determined using an unpaired two-tailed Student’s *t*-test.

DCFH-DA fluorescent probe was used to determine the intracellular ROS accumulation in *E. coli* B2 following OM19r combined with GEN treatment. The intracellular ROS accumulation of *E. coli* B2 was significantly increased (*p* < 0.01) in OM19r and OM19r combined with GEN treated groups compared with the control group (Figure 8). This suggests that OM19r treatment accumulated intracellular ROS, but gentamicin alone had no effect.

### 3.11. DNA binding assay

**Figure 9:**
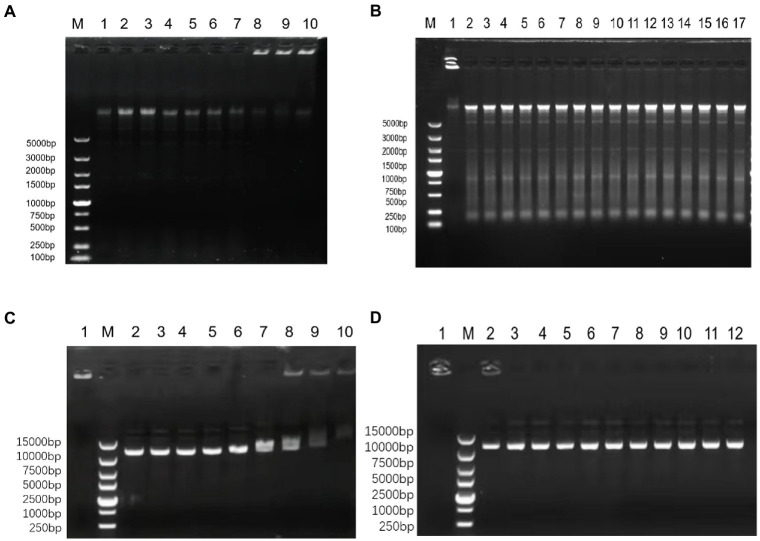
The effect of OM19r combined with GEN on the DNA of *E. coli* determined by DNA binding assay. **(A)** The effect of different concentrations of OM19r on the genomic DNA of *E. coli* B2, M: DL5000Marker; 1: *E. coli* B2 genome; 2: OM19r (2 μg/ml); 3: OM19r (4 μg/ml); 4: OM19r (8 μg/ml); 5: OM19r (16 μg/ml); 6: OM19r (32 μg/ml); 7: OM19r (64 μg/ml); 8: OM19r (128 μg/ml); 9:OM19r (256 μg/ml); 10: Positive control. **(B)** Effects of different concentrations of GEN and OM19r combined with GEN on the genomic DNA of *E. coli* B2. M: DL5000Marker; 1: Positive control 2: GEN (512 μg/ml); 3: GEN (256 μg/ml); 4: GEN (128 μg/ml); 5: GEN (64 μg/ml); 6: GEN (32 μg/ml); 7: GEN (16 μg/ml); 8: GEN (8 μg/ml); 9: GEN (4 μg/ml); 10: GEN (2 μg/ml); 11: GEN (1 μg/ml); 12: OM19r combined with GEN (2 + 1 μg/ml); 13: OM19r combined with GEN (2 + 2 μg/ml); 14: OM19r combined with GEN (4 + 2 μg/ml); 15: OM19r combined with GEN (4 + 4 μg/ml);16: OM19r combined with GEN (8 + 4 μg/ml);17:*E. coli* B2 genome. **(C)** The effects of different concentrations of OM19r on plasmid, 1: Positive control; M: 15000 DNA Marker; 2: plasmid; 3: OM19r (2 μg/ml); 4: OM19r (4 μg/ml); 5: OM19r (8 μg/ml); 6: OM19r (16 μg/ml); 7: OM19r (32 μg/ml);8: OM19r (64 μg/ml); 9: OM19r (128 μg/ml); 10: OM19r (256 μg/ml); **(D)** Effects of different concentrations of GEN and OM19r combined with GEN on plasmid. 1: Positive control; M: 15000 DNA Marker; 2: GEN (512 μg/ml); 3: GEN (256 μg/ml);4: GEN (128 μg/ml); 5: GEN (64 μg/ml); 6: GEN (32 μg/ml); 7: OM19r combined with GEN (2 + 1 μg/ml); 8: OM19r combined with GEN (2 + 2 μg/ml); 9: OM19r combined with GEN (4 + 2 μg/ml); 10: OM19r combined with GEN (4 + 4 μg/ml);11: OM19r combined with GEN (8 + 4 μg/ml); 12: plasmid.

OM19r (≥128 μg/ml) bound to *E. coli* B2 genomic DNA (Figures 9A–B), and OM19r (≥64 μg/ml) attached to the plasmid DNA (Figures 9C,D). However, OM19r combined with GEN (8 + 4 μg/ml) had no binding effect on *E. coli* B2 genomic DNA and plasmid DNA. These results indicated that the bacterial genomic DNA was not the target for OM19r combined with GEN.

### 3.12. *In vitro* protein synthesis

**Figure 10:**
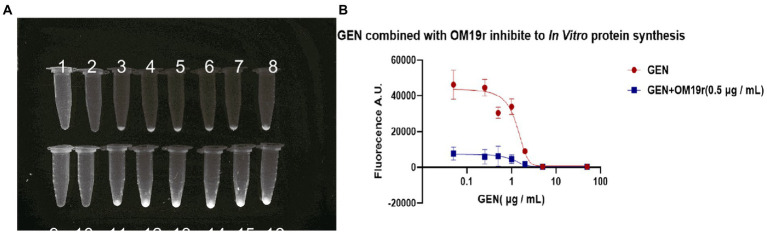
The effect of OM19r combined with GEN on *in vitro* protein synthesis. **(A)** EGFP green fluorescence observed under UV light by different concentrations of GEN and OM19r(0.5 μg/ml) combined with GEN for 16 h. 1: OM19r combined with GEN (50 μg/ml), 2: OM19r combined with GEN (5 μg/ml), 3: OM19r combined with GEN (2 μg/ml), 4: OM19r combined with GEN (1 μg/ml), 5: OM19r combined with GEN (0.5 μg/ml), 6: OM19r combined with GEN (0.25 μg/ml), 7: OM19r combined with GEN(0.05 μg/ml), 8: OM19r (0.5 μg/ml), 9: GEN (50 μg/ml), 10: GEN (5 μg/ml), 11: GEN (2 μg/ml), 12: GEN (1 μg/ml), 13: GEN (0.5 μg/ml), 14: GEN (0,25 μg/ml), 15: GEN (0.05 μg/ml), 16: negative control. **(B)** The fluorescence value of EGFP induced by the GEN and OM19r (0.5 μg/ml). Graphs show mean of three biological replicates.

EGFP green fluorescence was observed under UV light after the reaction of GEN and OM19r with the mixture at 29°C for 16 h. The results showed that the fluorescence intensity of EGFP decreased with the increase in GEN concentration (Figure 10A). These results suggested that GEN can inhibit EGFP expression *in vitro*. However, when OM19r (0.5 μg/ml) was added to each group, the fluorescence value of EGFP decreased significantly more than that GEN alone (Figure 10B). The above results indicated that OM19r alone could affect the synthesis of EGFP fluorescent protein.

### 3.13. *In vitro* transcription

**Figure 11:**
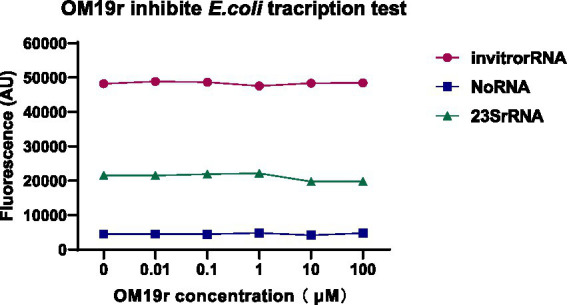
The effect of OM19r on the transcription process of *E. coli* determined by constructing the transcription system *in vitro*. Graphs show mean of three biological replicates.

The effect of OM19r on bacterial transcription was detected *in vitro*. The molecular beacon fluorescence signal of *E. coli* transcribed *in vitro* remained unchanged following treatment with OM19r, suggesting that OM19r had no effect on the transcription of *E. coli in vitro* (Figure 11).

### 3.14. mRNA translation

**Figure 12:**
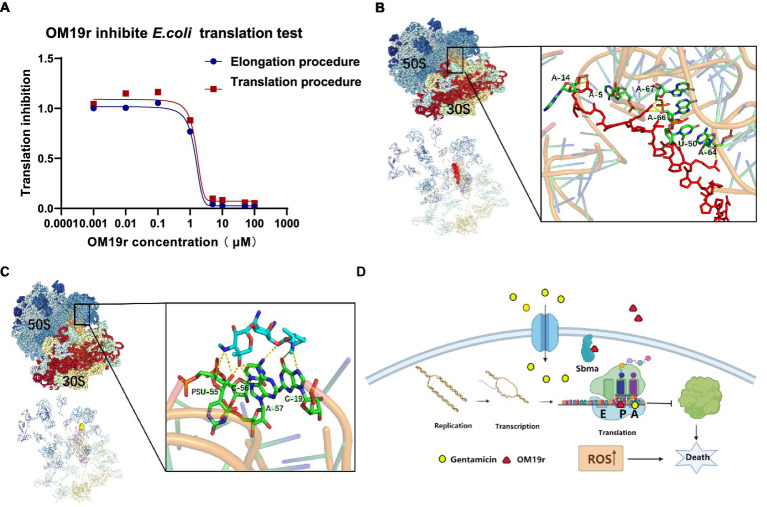
Translation elongation process of OM19r-targeted mRNA. **(A)** Dose–response of OM19r inhibition mRNA translation (red) and fMet-Phe dipeptide formation (blue) *in vitro*. **(B)** Molecular docking results of OM19r and ribosome. **(C)** Molecular docking results of GEN and ribosome. **(D)** Schematic diagram of synergistic antibacterial action mechanism of OM19r combined with GEN.

The dose–response curves for OM19r showed the inhibition of mRNA translation and Poly (U) formation overlap (Figure 12A), suggesting that inactivation of peptidyl transferase center (PTC) was caused by OM19r blocking mRNA translation. OM19r appeared to be an inhibitor of PTC activity, effectively blocking PTC specifically from producing the “first peptide bond” and mRNA translation. This may explain why the dose–response curves for inhibition of mRNA translation and Poly (U) formation were essentially overlapped. The molecular docking revealed that OM19r and GEN bind to ribosomes at different sites. The binding energy of OM19r to the ribosome was −115.33 kcal/mol (Supplementary Table S7). OM19r was predicted to bind to the ribosome’s A-5, A-14, A-64, A-66, A-67 and U-50 sites (Figure 12B). The binding energy of GEN to the ribosome was −140.8 kcal/mol (Supplementary Table S7). GEN was predicted to bind to the ribosome’s C-56, A-57, and G-19 sites (Figure 12C), proposing that OM19r entered the bacteria *via* SbmA and influenced the translation elongation process and ROS accumulation (Figure 12D).

### 3.15. Animal infection model

**Figure 13:**
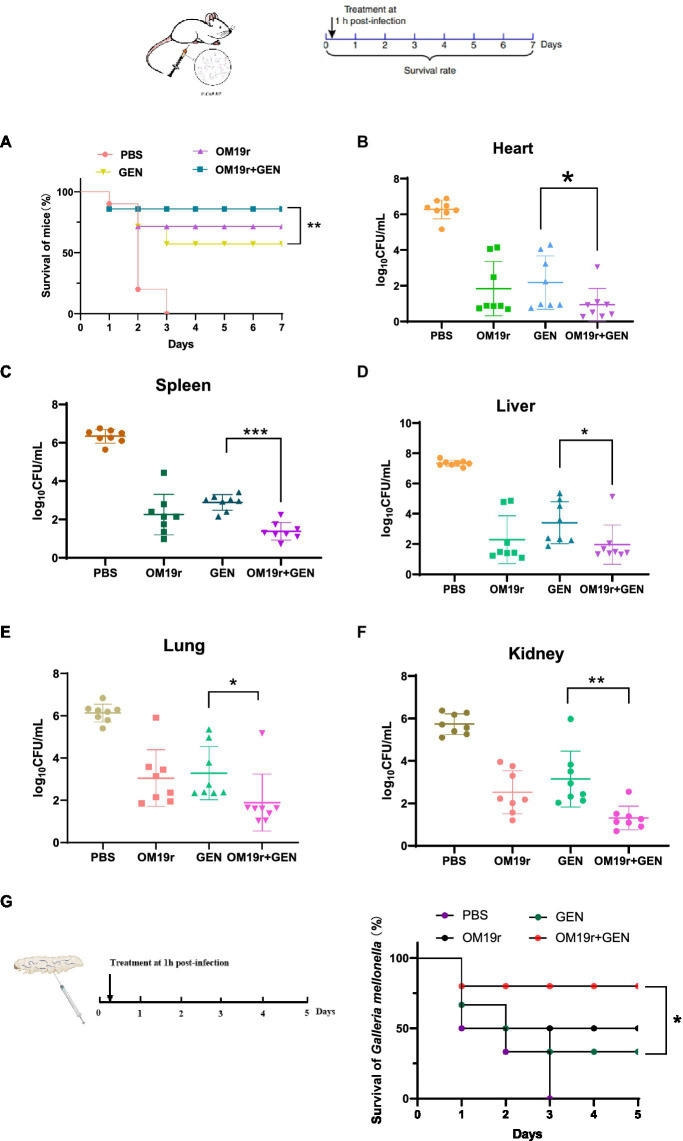
OM19r rescues GEN activity in two animal models of infection. **(A)** Survival curve of mice infected with non-lethal dose of *E. coli* B2 (3 × 10^8^ cfu/ml) for 7 days. *p*-values determined using a two-sided log[rank] (Mantel-Cox) test. **(B–F)** Bacterial load in all organs (heart, spleen, liver, lung and kidney) of mice measured after OM19r combined with GEN treatment. G, Survival curve of *G. mellonella* larvae infected with a non-lethal dose of *E. coli* B2 (1× 10^5^ cfu/ml) for 5 days. p-values determined using a two-sided log[rank] (Mantel-Cox) test.

OM19r restored susceptibility of drug-resistant strains to GEN, which was assessed in animal models infected with *E. coli* B2 (Figure 13). In mouse model of peritonitis, 80% of mice survived within 7 days of treatment with OM19r combined with GEN (4 + 2 mg/kg), significantly higher(*p* < 0.01) than the GEN (8 mg/kg) treated group (Figure 13A). The bacterial load test results showed that OM19r combined with GEN (4 + 2 mg/kg) significantly reduced the bacterial content in mice. The bacterial content in the liver, spleen, lung, and kidney was reduced by five-, four-, four-, and five-fold, respectively (Figures 13B–F). In *Galleria mellonella* (*G. mellonella*) larval model, 80% of *G. mellonella* survived within 7 days of treatment with OM19r combined with GEN (4 + 2 mg/kg), significantly higher (*p* < 0.01) than the GEN (8 mg/kg, 25% survival) treated group (Figure 13G). These findings suggested that LRGG is an antibiotic potentiator to enhance the antibacterial activity of GEN against *E. coil* B2 *in vivo*.

## 4. Discussion

Antibacterial synergists when used in combination with antibiotics could restore the sensitivity of drug-resistant pathogens up to some extent and prolong the effective life of antibiotics by reducing the resistance. Although there have been reports that AMPs used as synergists can enhance the antibacterial activity of antibiotics (Randhawa et al., 2016; Zhong et al., 2020), studies on antimicrobial peptides combined with gentamicin to reverse drug resistance in *E. coli* are lacking. Most AMPs contain cationic and amphiphilic molecules, which may be drawn to negatively charged cell membranes and damage the lipid bilayer (Shai, 2002). Therefore, AMPs usually exhibit low selectivity and broad-spectrum antibacterial activity against pathogenic bacteria (Matsuzaki, 2009). However, the antibacterial targets of some proline-rich antimicrobial peptides (PrAMPs) are often located inside the bacterial cells and do not cause obvious membrane damage (Nicolas, 2009; Scocchi et al., 2011, 2016; Graf et al., 2017). An antimicrobial peptide OM19r containing both proline and arginine was derived from the available peptide sequences in our laboratory. Compared with the broad-spectrum antimicrobial peptides, OM19r had a narrow antibacterial spectrum and antibacterial activity only against some Gram-negative bacteria. Meanwhile, OM19r had no combined antibacterial effect with other antibiotics at different action sites (FIC > 0.5), and OM19r reduced the MIC of GEN against multidrug-resistant *E. coli* B2 from 64 to 1 μg/ml. Some disadvantages of AMPs, such as hemolysis, cytotoxicity, and instability reported by other scientists hinder their clinical application (Kumar et al., 2017). In this study, the cytotoxicity and hemolytic activity of OM19r was measured *in vitro*. The results of safety assessment experiments at the cellular level indicated that OM19r had no hemolytic activity and cytotoxicity at OM19r ≤ 64 μg/ml. The results of animal infection model showed that OM19r restored GEN sensitivity of *E. coli* B2 *in vivo*. In conclusion, OM19r acted as a potential aminoglycoside antibiotic synergist.

The antibacterial mechanism of antimicrobial peptides involves membranous and non-membranous targeting. The antibacterial mechanism of OM19r on bacterial cell membranes was investigated *via* membrane permeability assay. The inner membrane permeability assays results showed that OM19r increased the inner membrane permeability of *E. coli* B2. SbmA as a member of the peptide uptake permease family (PUP) was located in some Gram-negative bacteria and played an important role in antibiotic transport (Paulsen et al., 2016). In previous studies, Oncocin was shown to enter the cell by the SbmA transporter. OM19r is an antimicrobial peptide heterozygous by Oncocin with the MDAP-2 fragment (Liu L. et al., 2020). So, the effect of OM19r on the bacterial inner membrane transporter SbmA protein was further determined by qPCR assays, SmbA protein was added *in vitro* and gene deletion strains were constrcuted. The qPCR results showed that mRNAs expression of SbmA was up-regulated following treatment with OM19r or OM19r combined with GEN. And the MIC of OM19r against *E. coli* B2 increased by adding purified SbmA protein *in vitro*. Meanwhile, SbmA gene deletion in *E. coli* ATTCC25922 resulted in the loss of the bacteriostatic activity of OM19r. It was further demonstrated that OM19r could enter the bacteria through membrane transporter SbmA. Molecular docking results showed that OM19r could bound to the ILE-47, ILE-217 and TYR-241 sites of SbmA. Finally, the CLSM and TEM results showed that OM19r entered *E. coli* B2 resulted in the release of the bacterial contents. In summary, OM19r was transported into *E. coli* B2 *via* SbmA to exert the bacteriostatic mechanism.

For non-membrane targeted antimicrobial peptides, current research on the mechanism has mainly focused on the effect of antimicrobial peptides on bacterial DNA (Bai et al., 2021) and protein levels (Roy et al., 2015). Therefore, the antibacterial mechanism of OM19r combined with GEN was studied in this study using DNA binding assay and cell-free expression assay. DNA binding test showed that OM19r could bind to *E. coli* genome at 128 μg/ml and *E. coli* plasmid at 64 μg/ml. GEN can bind to *E. coli* plasmid at concentration of 512 μg/ml. This indicate that high concentrations of OM19r and GEN (higher than the MIC) have binding effect on DNA. Therefore, OM19r combined with GEN mainly exerted antibacterial effects *via* mechanisms other than bacterial DNA. The effects of GEN and OM19r combined with GEN on protein synthesis were measured by fluorescence intensity of EGFP protein. GEN alone or OM19r combined with GEN affected the EGFP protein expression *in vitro.* Moreover, OM19r combined with GEN had a greater effect on the expression of EGFP protein than GEN alone. GEN influences the initial phase of bacterial translation, leading to mRNA misreading, and might explain the effect of GEN on EGFP protein expression *in vitro*. In summary, these results suggest that OM19r had no effect on DNA, but can affect protein synthesis.

Transcription and translation are important stages of protein synthesis in cells (Marras et al., 2004). The stage at which a drug acts on bacterial transcription and translation can be measured by constructing transcriptional translation systems *in vitro* (Marras et al., 2004; Brandi et al., 2007). Therefore, a transcription and translation systems were established to determine the specific inhibitory mechanism of OM19r on protein synthesis *in vitro*. Firstly, we proved by *in vitro* transcription experiments that the peptide has no effect on the transcription process. Secondly, since the *in vitro* protein synthesis could be inhibited by OM19r, but having no effect on RNA synthesis. Therefore, we focused on the protein translation process *in vitro*. A series of experiments was conducted and the results showed that OM19r had inhibition activity on the translation elongation process. Finally, molecular docking results further demonstrated that OM19r and GEN binds to different sites in the ribosome, with OM19r inhibiting the extension phase of protein translation and GEN inhibiting the initiation phase of translation.

Meanwhile, ROS has been reported to play a key role in bacterial resistance and the bactericidal activity of antibiotics (Van Acker and Coenye, 2017; Hong et al., 2019). Non-lethal concentrations of antibiotics cause ROS produced by bacteria, which can promote the SOS-DNA damage repair system, activate the stress protection mechanism, and form drug-resistant bacteria. Conversely, ROS produced by bacteria with lethal concentration of antibiotics can further accelerate the death of pathogens (Kohanski et al., 2010). The OM19r combined with GEN caused ROS accumulation in *E. coli* B2. In conclusion, OM19r can enter the cell through the intimal transporter SbmA, causing ROS accumulation in bacteria and inhibiting the translation extension stage of the protein, and ultimately causing death of the pathogen.

## 5. Conclusion

This study showed that OM19r combined with GEN had a strong synergistic inhibitory effect (FIC = 0.156) against multi-drug resistant *E. coli* B2. The combined inhibitory mechanism of OM19r and GEN shown that OM19r entered bacterial cells through SbmA transporter on cell membranes, thus causing intracellular ROS accumulation. Simultaneously, OM19r and GEN inhibited translation elongation and initiation, respectively, and ultimately affected the normal protein synthesis of bacteria. Two animal models shown that OM19r can also restore the sensitivity of multidrug-resistant *E. coli* B2 to GEN in animals. These findings offer a potential treatment option for the infections caused globally by multidrug-resistant *E. coli*.

## Data availability statement

The original contributions presented in the study are included in the article/Supplementary material, further inquiries can be directed to the corresponding authors.

## Ethics statement

The animal study was reviewed and approved by Jilin Agricultural University guidelines.

## Author contributions

H-XM, L-CK, and C-GH conceived and designed research. Q-JX, H-DY, YL, AG, AF, and X-YJ conducted experiments. H-PZ and P-HL contributed new reagents or analytical tools. Q-JX analyzed data. QC wrote the manuscript. All authors read and approved the manuscript.

## Funding

This study was funded by National Natural Science Foundation of China (31872519) and Jilin Scientific and Technological Development Program (20210202033NC, 20220508049RC, YDZJ202203CGZH050, 20230402037GH).

## Conflict of interest

The authors declare that the research was conducted in the absence of any commercial or financial relationships that could be construed as a potential conflict of interest.

## Publisher’s note

All claims expressed in this article are solely those of the authors and do not necessarily represent those of their affiliated organizations, or those of the publisher, the editors and the reviewers. Any product that may be evaluated in this article, or claim that may be made by its manufacturer, is not guaranteed or endorsed by the publisher.

## Footnotes

^1^ https://swissmodel.expasy.org/

## References

[ref1] Armas F. di Stasi A. Mardirossian M. Romani A. A. Benincasa M. Scocchi M. (2021). Effects of Lipidation on a proline-rich antibacterial peptide. Int. J. Mol. Sci. 22:7959. doi: 10.3390/ijms22157959, PMID: 34360723 PMC8347091

[ref2] Aslam B. Wang W. Arshad M. I. Khurshid M. Muzammil S. Rasool M. H.. (2018). Antibiotic resistance: a rundown of a global crisis. Infect. Drug Resist. 11, 1645–1658. doi: 10.2147/IDR.S173867, PMID: 30349322 PMC6188119

[ref3] Bai S. Wang J. Yang K. Zhou C. Xu Y. Song J.. (2021). A polymeric approach toward resistance-resistant antimicrobial agent with dual-selective mechanisms of action. Sci. Adv. 7:eabc9917. doi: 10.1126/sciadv.abc9917, PMID: 33571116 PMC7840121

[ref4] Böttger R. Knappe D. Hoffmann R. (2016). Readily adaptable release kinetics of prodrugs using protease-dependent reversible PEGylation. J. Control. Release 230, 88–94. doi: 10.1016/j.jconrel.2016.04.010 27067364

[ref5] Brandi L. Fabbretti A. Milon P. Carotti M. Pon C. L. Gualerzi C. O. (2007). Methods for identifying compounds that specifically target. Methods Enzymol. 431, 229–267. doi: 10.1016/S0076-6879(07)31012-4 17923238

[ref6] Bustin S. A. Benes V. Garson J. A. Hellemans J. Huggett J. Kubista M.. (2009). The MIQE guidelines: minimum information for publication of quantitative real-time PCR experiments. Clin. Chem. 55, 611–622. doi: 10.1373/clinchem.2008.112797 19246619

[ref7] Corbalan N. Runti G. Adler C. Covaceuszach S. Ford R. C. Lamba D.. (2013). Functional and structural study of the dimeric inner membrane protein SbmA. J. Bacteriol. 195, 5352–5361. doi: 10.1128/JB.00824-13, PMID: 24078611 PMC3837955

[ref8] Cui Q. Qi-Jun X. Lei L. Li-Li G. Xiu-Yun J. Muhammad I.. (2021). Preparation, characterization and pharmacokinetic study of N-terminal PEGylated D-form antimicrobial peptide OM19r-8. J. Pharm. Sci. 110, 1111–1119. doi: 10.1016/j.xphs.2020.10.048, PMID: 33129837

[ref9] Ding X. Yang C. Moreira W. Yuan P. Periaswamy B. Sessions P. F.. (2020). A macromolecule reversing antibiotic resistance phenotype and repurposing drugs as potent antibiotics. Adv. Sci. 7:2001374. doi: 10.1002/advs.202001374, PMID: 32995131 PMC7503100

[ref10] Durand-Reville T. F. Miller A. A. O'Donnell J. P. Wu X. Sylvester M. A. Guler S.. (2021). Rational design of a new antibiotic class for drug-resistant infections. Nature 597, 698–702. doi: 10.1038/s41586-021-03899-0, PMID: 34526714

[ref11] Falciani C. Zevolini F. Brunetti J. Riolo G. Gracia R. Marradi M.. (2020). Antimicrobial peptide-loaded nanoparticles as inhalation therapy for *Pseudomonas aeruginosa* infections. Int. J. Nanomedicine 15, 1117–1128. doi: 10.2147/IJN.S218966 32110011 PMC7034994

[ref12] Garamella J. Marshall R. Rustad M. Noireaux V. (2016). The all E. coli TX-TL toolbox 2.0: a platform for cell-free synthetic biology. ACS Synth. Biol. 5, 344–355. doi: 10.1021/acssynbio.5b00296, PMID: 26818434

[ref13] Graf M. Mardirossian M. Nguyen F. Seefeldt A. C. Guichard G. Scocchi M.. (2017). Proline-rich antimicrobial peptides targeting protein synthesis. Nat. Prod. Rep. 34, 702–711. doi: 10.1039/c7np00020k, PMID: 28537612

[ref14] Hong Y. Zeng J. Wang X. Drlica K. Zhao X. (2019). Post-stress bacterial cell death mediated by reactive oxygen species. Proc. Natl. Acad. Sci. U. S. A. 116, 10064–10071. doi: 10.1073/pnas.1901730116, PMID: 30948634 PMC6525477

[ref15] Hooban B. Fitzhenry K. O'Connor L. Miliotis G. Joyce A. Chueiri A.. (2022). A longitudinal survey of antibiotic-resistant Enterobacterales in the Irish environment, 2019-2020. Sci. Total Environ. 828:154488. doi: 10.1016/j.scitotenv.2022.154488, PMID: 35278563

[ref16] Jeamsripong S. Li X. Aly S. S. Su Z. Pereira R. V. Atwill E. R. (2021). Antibiotic resistance genes and associated phenotypes in *Escherichia coli* and *Enterococcus* from cattle at different production stages on a dairy farm in Central California. Antibiotics (Basel) 10:1042. doi: 10.3390/antibiotics10091042, PMID: 34572624 PMC8471271

[ref17] Jia B. Y. Wang Y. M. Zhang Y. Wang Z. Wang X. Muhammad I.. (2020). High cell selectivity and bactericidal mechanism of symmetric peptides centered on d-pro-Gly pairs. Int. J. Mol. Sci. 21:1140. doi: 10.3390/ijms21031140, PMID: 32046328 PMC7037546

[ref18] Kaminishi T. Schedlbauer A. Fabbretti A. Brandi L. Ochoa-Lizarralde B. He C. G.. (2015). Crystal lographic characterization of the ribosomal binding site and molecular mechanism of action of Hygromycin a. Nucleic Acids Res. 43, 10015–10025. doi: 10.1093/nar/gkv975, PMID: 26464437 PMC4787777

[ref20] Kohanski M. DePristo M. A. Collins J. J. (2010). Sublethal antibiotic treatment leads to multidrug resistance via radical-induced mutagenesis. Mol. Cell 37, 311–320. doi: 10.1016/j.molcel.2010.01.003, PMID: 20159551 PMC2840266

[ref21] Kumar A. Mahajan M. Awasthi B. Tandon A. Harioudh M. K. Shree S.. (2017). Piscidin-1-analogs with double L- and D-lysine residues exhibited different conformations in lipopolysaccharide but comparable anti-endotoxin activities. Sci. Rep. 7:39925. doi: 10.1038/srep39925, PMID: 28051162 PMC5209718

[ref22] Liu Y. Jia Y. Yang K. Li R. Xiao X. Zhu K.. (2020). Metformin restores Tetracyclines susceptibility against multidrug resistant bacteria. Adv. Sci. (Weinh) 7:1902227. doi: 10.1002/advs.201902227, PMID: 32596101 PMC7312304

[ref23] Liu L. Liu J. Cui Q. Jia B. Y. Ma H. X. (2020). Design and characterization of a novel hybrid antimicrobial peptide om19r based on oncocin and mdap-2. Int. J. Pept. Res. Ther. 26, 1839–1846. doi: 10.1007/s10989-019-09984-3

[ref24] Ma L. Xie X. Liu H. Huang Y. Wu H. Jiang M.. (2020). Potent antibacterial activity of MSI-1 derived from the magainin 2 peptide against drug-resistant bacteria. Theranostics 10, 1373–1390. doi: 10.7150/thno.39157, PMID: 31938070 PMC6956804

[ref001] Mans R. J. Novelli G. D. (1961). Measurement of the incorporation of radioactive amino acids into protein by a filter-paper disk method. Archives of Biochemistry and Biophysics. 94, 48–53. doi: 10.1016/0003-9861(61)90009-1

[ref25] Maisuria V. B. Hosseinidoust Z. Tufenkji N. (2015). Polyphenolic extract from maple syrup potentiates antibiotic susceptibility and reduces biofilm formation of pathogenic bacteria. Appl. Environ. Microbiol. 81, 3782–3792. doi: 10.1128/AEM.00239-15, PMID: 25819960 PMC4421064

[ref26] Marras S. A. Gold B. Kramer F. R. Smith I. Tyagi S. (2004). Real-time measurement of *in vitro* transcription. Nucleic Acids Res. 32:e72. doi: 10.1093/nar/gnh068, PMID: 15155820 PMC419623

[ref27] Matsuzaki K. (2009). Control of cell selectivity of antimicrobial peptides. Biochim. Biophys. Acta 1788, 1687–1692. doi: 10.1016/j.bbamem.2008.09.013 18952049

[ref28] Mattiuzzo M. Bandiera A. Gennaro R. Benincasa M. Pacor S. Antcheva N.. (2010). Role of the *Escherichia coli* SbmA in the antimicrobial activity of proline-rich peptides. Mol. Microbiol. 66, 151–163. doi: 10.1111/j.1365-2958.2007.05903.x, PMID: 17725560

[ref29] Miao X. Zhou T. Zhang J. Xu J. Guo X. Hu H.. (2020). Enhanced cell selectivity of hybrid peptides with potential antimicrobial activity and immunomodulatory effect. Biochim. Biophys. Acta Gen. Subj. 1864:129532. doi: 10.1016/j.bbagen.2020.129532, PMID: 31953126

[ref30] Mwangi J. Yin Y. Wang G. Yang M. Li Y. Zhang Z.. (2019). The antimicrobial peptide ZY4 combats multidrug-resistant Pseudomonas aeruginosa and *Acinetobacter baumannii* infection. Proc. Natl. Acad. Sci. U. S. A. 116, 26516–26522. doi: 10.1073/pnas.1909585117, PMID: 31843919 PMC6936460

[ref31] Nicolas P. (2009). Multifunctional host defense peptides: intracellular-targeting antimicrobial peptides. FEBS J. 276, 6483–6496. doi: 10.1111/j.1742-4658.2009.07359.x, PMID: 19817856

[ref32] Paulsen V. S. Mardirossian M. Blencke H. M. Benincasa M. Runti G. Nepa M.. (2016). Inner membrane proteins YgdD and SbmA are required for the complete susceptibility of *E. coli* to the proline-rich antimicrobial peptide arasin 1(1-25). Microbiology 162, 601–609. doi: 10.1099/mic.0.000249 26860543

[ref33] Peng T. Huiyang F. Xi M. (2021). Design, optimization, and nanotechnology of antimicrobial peptides: from exploration to applications. Nano Today 39:101229. doi: 10.1016/j.nantod.2021.101229

[ref34] Randhawa H. K. Gautam A. Sharma M. Bhatia R. Varshney G. C. Raghava G. P.. (2016). Cell-penetrating peptide and antibiotic combination therapy: a potential alternative to combat drug resistance in methicillin-resistant *Staphylococcus aureus*. Appl. Microbiol. Biotechnol. 100, 4073–4083. doi: 10.1007/s00253-016-7329-7, PMID: 26837216

[ref35] Roy R. N. Lomakin I. B. Gagnon M. G. Steitz T. A. (2015). The mechanism of inhibition of protein synthesis by the proline-rich peptide oncocin. Nat. Struct. Mol. Biol. 22, 466–469. doi: 10.1038/nsmb.3031, PMID: 25984972 PMC4456192

[ref36] Scocchi M. Mardirossian M. Runti G. Benincasa M. (2016). Non-membrane Permeabilizing modes of action of antimicrobial peptides on bacteria. Curr. Top. Med. Chem. 16, 76–88. doi: 10.2174/1568026615666150703121009, PMID: 26139115

[ref37] Scocchi M. Tossi A. Gennaro R. (2011). Proline-rich antimicrobial peptides: converging to a non-lytic mechanism of action. Cell. Mol. Life Sci. 68, 2317–2330. doi: 10.1007/s00018-011-0721-7, PMID: 21594684 PMC11114787

[ref38] Shai Y. (2002). Mode of action of membrane active antimicrobial peptides. Biopolymers 66, 236–248. doi: 10.1002/bip.10260 12491537

[ref39] Si Z. Lim H. W. Tay M. Y. F. Du Y. Ruan L. Qiu H.. (2020). A glycosylated cationic block poly(β-peptide) reverses intrinsic antibiotic resistance in all ESKAPE gram-negative bacteria. Angew. Chem. Int. Ed. Engl. 59, 6819–6826. doi: 10.1002/anie.201914304, PMID: 32011781

[ref40] Song M. Liu Y. Huang X. Ding S. Wang Y. Shen J.. (2020). A broad-spectrum antibiotic adjuvant reverses multidrug-resistant gram-negative pathogens. Nat. Microbiol. 5, 1040–1050. doi: 10.1038/s41564-020-0723-z 32424338

[ref41] Song M. Liu Y. Li T. Liu X. Hao Z. Ding S.. (2021). Plant natural flavonoids against multidrug resistant pathogens. Adv. Sci. 8:e2100749:2100749. doi: 10.1002/advs.202100749, PMID: 34041861 PMC8336499

[ref42] Stokes J. M. MacNair C. R. Ilyas B. French S. Côté J. P. Bouwman C.. (2017). Pentamidine sensitizes gram-negative pathogens to antibiotics and overcomes acquired colistin resistance. Nat. Microbiol. 2:17028. doi: 10.1038/nmicrobiol.2017.28, PMID: 28263303 PMC5360458

[ref43] Sturge C. R. Felder-Scott C. F. Pifer R. Pybus C. Jain R. Geller B. L.. (2019). AcrAB-TolC inhibition by peptide-conjugated Phosphorodiamidate Morpholino oligomers restores antibiotic activity in vitro and in vivo. ACS Infect. Dis. 5, 1446–1455. doi: 10.1021/acsinfecdis.9b00123, PMID: 31119935

[ref44] Türkez H. Enes A. M. Erdal S. Fatime G. Metin A. Abdulgani T. (2019). Microarray assisted toxicological investigations of boron carbide nanoparticles on human primary alveolar epithelial cells. Chem. Biol. Interact. 300, 131–137. doi: 10.1016/j.cbi.2019.01.021, PMID: 30684454

[ref45] Van Acker H. Coenye T. (2017). The role of reactive oxygen species in antibiotic-mediated killing of bacteria. Trends Microbiol. 25, 456–466. doi: 10.1016/j.tim.2016.12.008, PMID: 28089288

[ref46] Van Boeckel T. P. Pires J. Silvester R. Zhao C. Song J. Criscuolo N. G.. (2019). Global trends in antimicrobial resistance in animals in low- and middle-income countries. Science 365:eaaw1944:365. doi: 10.1126/science.aaw1944, PMID: 31604207

[ref47] Wang J. Chou S. Xu L. Zhu X. Dong N. Shan A.. (2015). High specific selectivity and membrane-active mechanism of the synthetic centrosymmetric α-helical peptides with Gly-Gly pairs. Sci. Rep. 5:15963. doi: 10.1038/srep15963, PMID: 26530005 PMC4632126

[ref48] Wang J. Dou X. Song J. Lyu Y. Zhu X. Xu L.. (2019). Antimicrobial peptides: promising alternatives in the post feeding antibiotic era. Med. Res. Rev. 39, 831–859. doi: 10.1002/med.21542, PMID: 30353555

[ref49] Xu L. Shao C. Li G. Shan A. Chou S. Wang J.. (2020). Conversion of broad-Spectrum antimicrobial peptides into species-specific antimicrobials capable of precisely targeting pathogenic bacteria. Sci. Rep. 10:944. doi: 10.1038/s41598-020-58014-6, PMID: 31969663 PMC6976587

[ref50] Zheng Z. Tharmalingam N. Liu Q. Jayamani E. Kim W. Fuchs B. B.. (2017). Synergistic efficacy of Aedes aegypti antimicrobial peptide Cecropin A2 and tetracycline against Pseudomonas aeruginosa. Antimicrob. Agents Chemother. 61, e00686–e00617. doi: 10.1128/AAC.00686-17, PMID: 28483966 PMC5487646

[ref51] Zhong C. Gou S. Liu T. Zhu Y. Zhu N. Liu H.. (2019). Study on the effects of different dimerization positions on biological activity of partial d-amino acid substitution analogues of Anoplin. Microb. Pathog. 139:103871. doi: 10.1016/j.micpath.2019.103871, PMID: 31733278

[ref52] Zhong C. Zhang F. Zhu N. Zhu Y. Yao J. Gou S.. (2021). Ultra-short lipopeptides against gram-positive bacteria while alleviating antimicrobial resistance. Eur. J. Med. Chem. 212:113138. doi: 10.1016/j.ejmech.2020.113138, PMID: 33422980

[ref53] Zhong C. Zhu N. Zhu Y. Liu T. Gou S. Xie J.. (2020). Antimicrobial peptides conjugated with fatty acids on the side chain of D-amino acid promises antimicrobial potency against multidrug-resistant bacteria. Eur. J. Pharm. Sci. 141:105123. doi: 10.1016/j.ejps.2019.105123, PMID: 31676352

[ref54] Zhu N. Zhong C. Liu T. Zhu Y. Gou S. Bao H.. (2021). Newly designed antimicrobial peptides with potent bioactivity and enhanced cell selectivity prevent and reverse rifampin resistance in gram-negative bacteria. Eur. J. Pharm. Sci. 158:105665. doi: 10.1016/j.ejps.2020.105665, PMID: 33285267

