# Supplementary material for: Antibiotic synergist OM19r reverses aminoglycoside resistance in multidrug-resistant Escherichia coli
Source: Front Microbiol. 2023 Apr 18;14:1144946. doi: 10.3389/fmicb.2023.1144946 (PMC10151501; doi:10.3389/fmicb.2023.1144946)
Supplement: Supplementary file 1 [file Data_Sheet_1.pdf]

Supplementary Material

Antibiotic synergist OM19r Reverses Aminoglycoside Resistance in Multidrug-Resistant *Escherichia coli*

Qi Cui[1†], Han-Dong Yu[2†], Qi-Jun Xu[2], Yue Liu[1], Yu-Ting Wang[1], Peng-Hui Li[2], Ling-Cong Kong[2], Hai-Peng Zhang[1], Xiu-Yun Jiang[1], Anna Maria Giuliodori[3], Attilio Fabbretti[3], Cheng-Guang He[1*] and Hong-Xia Ma[1,2*]

* **Correspondence:** Hong-Xia Ma: hongxia0731001@163.com,

Cheng- Guang He: hechengguang@163.com

1 Supplementary Figures and Tables

1.1 Supplementary Figures

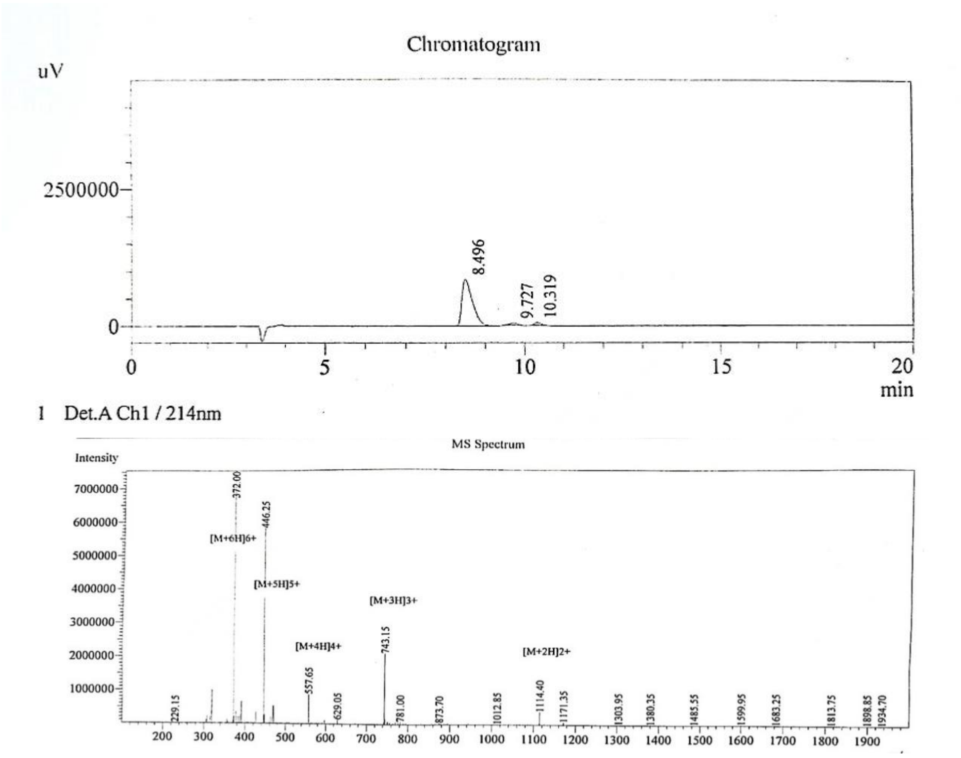

Figure. S1 The synthetic mass spectrum of antimicrobial peptide OM19r

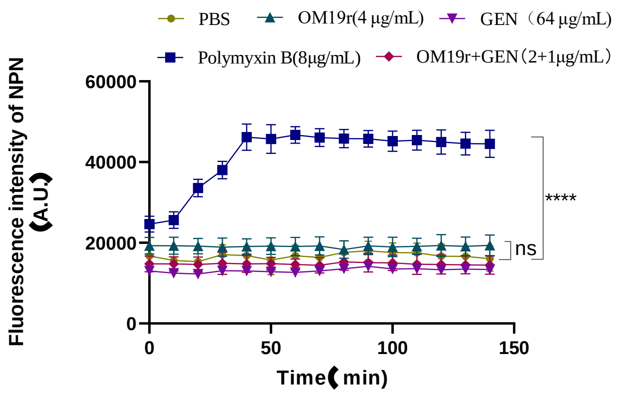

Figure. S2 Outer membrane permeability dynamics probed by NPN. All experiments were performed as three biologically independent experiments and mean ± SD are shown. P-values were determined using an unpaired two-tailed Student's t-test.

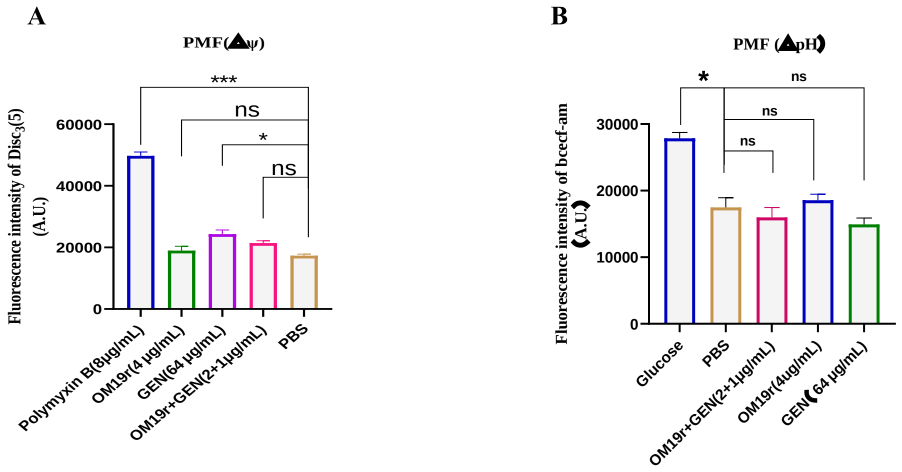

Figure S3. Effect of OM19r combined with GEN on PMF of *E. coli* B2. A, Dynamic changes in △φ of *E. coli* B2 were determined by 3, 3-dipropylthiodicyanide dis3 (5). B, Dynamic changes in △pH of *E. coli* B2 were determined by BCECF-AM. Graphs showed the mean of three biological replicates, P-values were determined using an unpaired two-tailed Student's t-test.

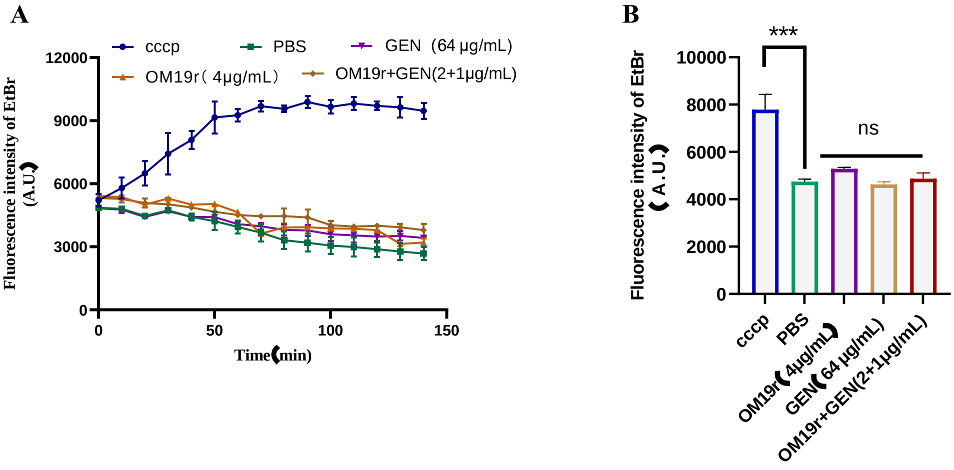

Figure S4. Effect of OM19r combined with GEN on Efflux pump of *E. coli* B2. A, Dynamic changes in Efflux pump of *E. coli* B2 were determined by EtBr, fluorescence intensity was detected every 10 minutes for 150 minutes. B, Dynamic changes in Efflux pump of *E. coli* B2 were determined by EtBr at 30 min. Graphs showed the mean of three biological replicates, P-values were determined using an unpaired two-tailed Student's t-test.

1.2 Supplementary Tables

Table S1.MIC of different antimicrobial peptides against *E. coli* (μg/mL)

Antimicrobial peptide	*E. coli* ATCC25922	*E. coli* B2
OM19r	1	4
OM19R	1	4
MB7R	2	4
MB19	2	4
LR_{PG}	2	8
LR_{GG}	2	4
LRα	2	4

Table S2.FIC of different antimicrobial peptides combined with gentamicin against *E. coli* B2(μg/mL)

Antimicrobial peptide	FIC
OM19r	0.156
OM19R	0.425
MB7R	1
MB19	1
LR_{PG}	0.5
LR_{GG}	0.5
LRα	0.375

Table S3. MIC of hybrid antimicrobial peptides against *E. coli* (μg/mL)

Antimicrobial peptide	Sequence	*E. coli* ATCC25922	*E. coli* B2
MDAP-2	SRDSRPVQPRVQPPPPPPKQKPSIYDT**PIRRPGGR**KTMYA	128	64
Oncocin	**VDKPPYLPRPR**PPRRIYNR-NH2	8	8
OM19R	VDKPPYLPRPRPIRRPGGR-NH2	1	4
OM19r	VDKPPYLPRPRPIRrPGGr-NH2	1	4

Table S4. Antibacterial effect of OM19r combined with 5 antibiotics on *E. coli* B2

Antibiotics	Action site	Combination MIC（μg/mL）	MIC Breakpoints（μg/mL）	Combined FIC
ceftriaxone	cell wall	256（R）	4	1
enrofloxacin	DNA gyrase	128（R）	1	1
tetracycline	30S ribosome	32（R）	16	0.5
rifampicin	RNA polymerase	64（R）	16	0.75
flufenicol	50S ribosome	64（R）	32	0.75
gentamicin	30S ribosome	2（S）	16	0.156

Note: R: resistant, S: sensitive

Table S5. Resistance spectrum of multidrug-resistant Gram-negative bacteria selected in this study

**Antibiotic**(μg/mL)	**Colistin**	**Enrofloxacin**	**Tetracycline**	**Florfenicol**	**Erythromycin**	**Kanamycin**	**Streptomycin**	**Tobramycin**	**Amikacin**	**Spectinomycin**	**Gentamicin**
E. coil ATCC 25922	0.25	1	1	2	64	1	4	1	1	1	0.25
E. coil B2	8	32	128	256	256	512	256	128	16	512	64
S. typhimurium 1A	64	32	16	8	512	256	2	8	2	256	512
A. baumannii JS1	0.015	256	512	128	64	64	512	64	32	512	512
S. dysenteriae A3	0.067	0.067	32	4	64	64	64	32	16	256	16
K. Pneumoniae JP20	1	128	512	256	128	>512	16	16	4	512	128

Table S6. Antibacterial effect of OM19r combined with different aminoglycosides against drug-resistant *E. coli* B2

Antibiotic	MIC[a] (μg/mL)	FIC index	MIC[b] with OM19r (μg/mL)	Potentiation（fold）[c]
kanamycin	512	0.375	2	256
gentamicin	64	0.157	1	64
streptomycin	256	0.188	2	128
tobramycin	64	0.375	4	64
spectinomycin	512	0.375	2	256

a/b MICs of antibiotic in the absence or presence of 0.5×MIC OM19r .

c Degree of antibiotic potentiation in the presence of 0.5×MIC OM19r.

Table S7. Molecular docking results

Antimicrobial agents	Receptor	Binding Energy (kcal/mol）	Ligand RMSD (A)
OM19r	SbmA	-262.92	204.09
OM19r	ribosome	-115.33	242.11
GEN	ribosome	-140.8	288.83
